# Carbon-monoxide-driven bioethanol production operates through a tungsten-dependent catalyst

**DOI:** 10.1038/s41589-025-02055-3

**Published:** 2025-10-29

**Authors:** Olivier N. Lemaire, Mélissa Belhamri, Anna Shevchenko, Tristan Wagner

**Affiliations:** 1 Max Planck Institute for Marine Microbiology, Bremen, Germany; 2 Max Planck Institute of Molecular Cell Biology and Genetics, Dresden, Germany; 3 Institut de Biologie Structurale, Université Grenoble Alpes, CEA, CNRS, Grenoble, France

**Keywords:** Enzymes, X-ray crystallography, Industrial microbiology, Metabolic pathways, Biocatalysis

## Abstract

Microbial alcohol production from waste gases is a game changer for sustainable carbon cycling and remediation. While the biotechnological process using *Clostridium* *autoethanogenum* to transform syngas (H_2_, CO_2_ and CO) is blooming, scientific debates remain on the ethanol biosynthesis pathway. Here, we experimentally validated that ethanol production is initiated through a tungsten-dependent aldehyde:ferredoxin oxidoreductase (AFOR), which reduces acetate to acetaldehyde. The reaction, thermodynamically unfavorable under standard conditions, has been considered by many as unsuitable in vivo but is rather approved by metabolic modeling. To answer this riddle, we demonstrated that the thermodynamic coupling of CO oxidation and ethanol synthesis allows acetate reduction. The experiments, performed with native CO dehydrogenase and AFOR, highlighted the key role of ferredoxin in stimulating the activity of both metalloenzymes and electron shuttling. The crystal structure of holo AFOR, refined to 1.59-Å resolution, and its biochemical characterization provide new insights into the cofactor chemistry and the specificities of this enzyme, fundamental to sustainable biofuel production.

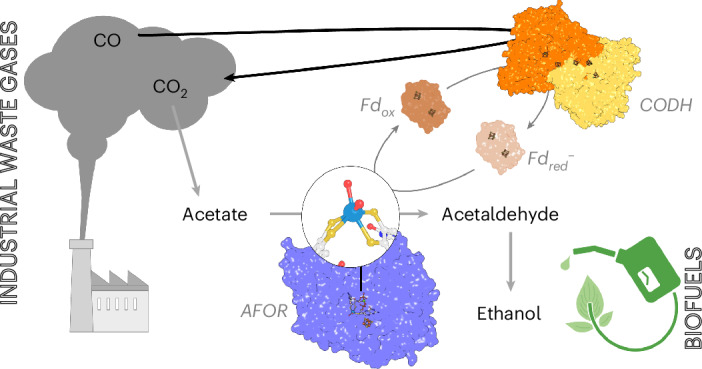

## Abstract (web summary)

Microbial alcohol production from waste gases could enable sustainable carbon cycling. Now, the tungsten-dependent aldehyde:ferredoxin oxidoreductase from the gas-converting bacterium *Clostridium* *autoethanogenum* has been biochemically and structurally characterized. This monomeric enzyme is responsible for acetate reduction to acetaldehyde, the key step of ethanol production in this industrial microorganism.

## Main

The transformation of synthetic gases into alcohols is a promising strategy for the carbon-cycling economy by converting the greenhouse gas CO_2_ and the polluting gas CO into added-value molecules. In that regard, *Clostridium* *autoethanogenum* is already widely used as a bioconverter in industry to turn concentrated waste gases (H_2_, CO_2_ and CO) into acetate, 2,3-butanediol and ethanol^1–8^. The scientific community still debates how ethanol production is orchestrated in such an organism. Following the most common hypothesis based on genetic and biochemical considerations, the pathway would start with the reduction of acetate by an aldehyde:ferredoxin oxidoreductase (AFOR) and the successive acetaldehyde reduction to ethanol by an alcohol dehydrogenase (ADH)^5,8–10^. The first and second reactions would be fueled by the oxidation of the reduced ferredoxin and NAD(P)H pools, respectively. One of the sources of reduced ferredoxin is CO oxidation catalyzed by the CO dehydrogenase–acetyl-coenzyme A (CODH/ACS) complex^6,11^. NAD(P)H is indirectly obtained through ferredoxin oxidation (NADH by the Rnf system and NADPH by the Nfn system)^5^.

Recent biochemical characterization of homologous AFOR challenged the plausible role of AFOR in bacterial ethanol production because of the unfavorable reaction of aldehyde generation. As with other acids, acetate reduction requires a very low redox potential (*E*^0^′ = −580 mV for the acetate/acetaldehyde couple), which would be challenging to perform when considering the standard redox potentials of electron donors available in living organisms^12–14^. The overall reaction would be highly endergonic with regular ferredoxin as an electron donor (considering a standard midpoint redox potential of *E*^0^′ = −400 mV)^15^. Therefore, it was proposed that AFOR homologs are physiologically involved in the thermodynamically favorable aldehyde oxidation rather than acid reduction^12–14^, suggesting that acetaldehyde could rather be produced by an alternative pathway. However, this statement is in direct contradiction with physiological studies, kinetic models and thermodynamics-based metabolic flux analysis in *C*. *autoethanogenum*, establishing AFOR as the central enzyme for ethanol production in the microorganism^16–19^. Several studies pointed out that the AFOR-dependent acid reduction to aldehyde in *C*. *autoethanogenum* could be thermodynamically favorable in vivo, directly refuting the thermodynamics argument against the AFOR role in ethanol production^5,17,18^.

To solve this metabolic riddle, we characterized the native AFOR of *C*. *autoethanogenum* (*Ca*AFOR) by X-ray crystallography, biochemistry and enzymology. On top of providing important information regarding the enzyme activity and mechanism, we showed that the ethanol production pathway is fueled by CO oxidation and favored by the immediate acetaldehyde consumption, sustaining a flux toward alcohol formation and maintaining low intracellular CO concentration.

## Results

### *Ca*AFOR is a tungsten-containing enzyme inactive as isolated

**Extended Data Fig. 1:**
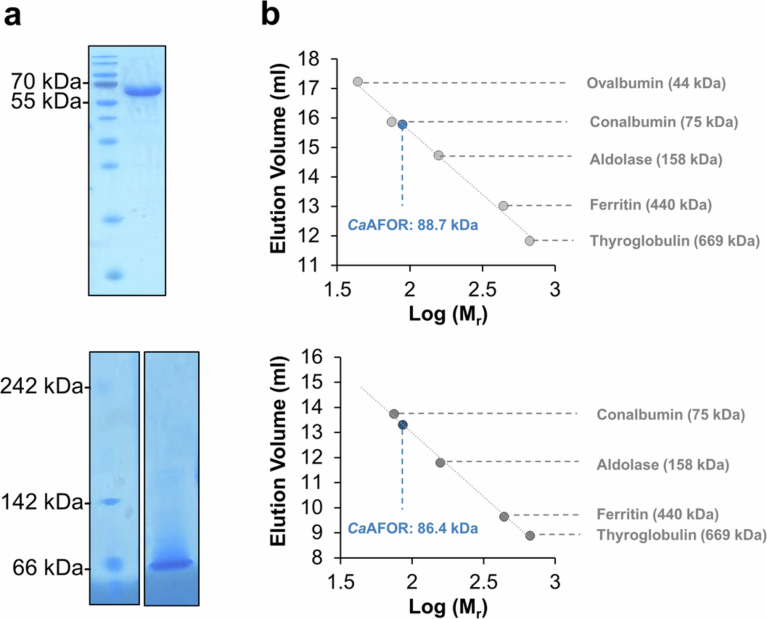
Size determination of the AFOR from *C. autoethanogenum.* **a**, Denaturing (top) and native (bottom) electrophoresis profile of the purified *Ca*AFOR (3 µg). **b**, Determination of molecular weight by size exclusion chromatography. The molecular weight of *Ca*AFOR (blue circles) was determined using a Superose 6 increase 10/300 GL (top), a Superdex 200 10/300 increase GL (bottom) and a protein standard calibration kit (gray circles). *Ca*AFOR has an expected molecular weight of 66,335 Da based on its sequence. The electrophoresis and size exclusion chromatography have been regularly performed on the enzymes obtained from different purifications (more than five times), with similar obtained results. Source data

**Extended Data Fig. 2:**
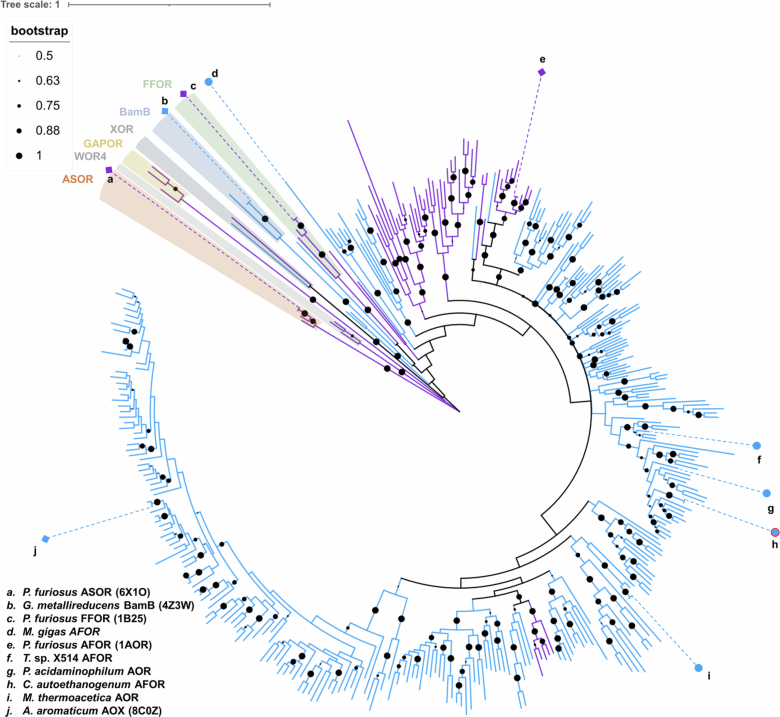
Phylogenetic tree of aldehyde oxidases. The maximum likelihood phylogenetic tree was built by using 369 sequences composed of (*i*) *Ca*AFOR homologues presenting 90% or less identity, (*ii*) sequences of the structurally characterised homologues, and (*iii*) sequences used in the phylogenetic analysis from Arndt et al. 2019^12^. Node scores were calculated on 200 replicates. Branches are coloured blue (bacteria) or purple (archaea), and enzyme types are indicated^12^. The closest branch of the characterised enzymes is indicated and labelled. Structurally characterised enzymes are indicated as squares, and biochemically characterised enzymes are coloured in circles depending on the taxonomy. The list of sequences used for the analysis is available on the public repository Zenodo under the accession ID 15229728 ref. ^61^.

**Extended Data Fig. 3:**
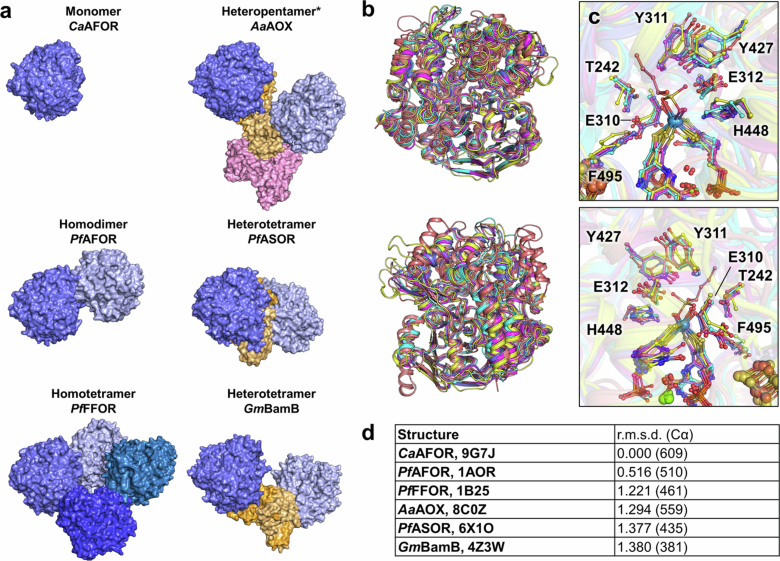
Structural organisation of aldehyde oxidases-containing complexes. **a**, Organisation of the structurally characterised aldehyde oxidases. Proteins are shown as surfaces with aldehyde oxidases, electron transfer subunits, and NAD^+^-reducing subunits in shades of blue, orange, and pink, respectively. The asterisk for the heteropentameric *Aa*AOX highlights the property of this complex to generate nanowire-like structures. **b**, **c**, Superposition of the aldehyde oxidases subunits. **b, c**, Proteins are shown as cartoon coloured as follows: *Ca*AFOR (9G7J, slate), the AFOR from *P. furiosus* (1AOR, pink), the FFOR from *P. furiosus* (1B25, cyan), the AOX from *A. aromaticum* (8C0Z, yellow), and the ASOR from *P. furiosus* (6X1O, salmon). Alignment root mean square deviation (r.m.s.d.) and aligned Cα are given in **d**. The numbering is from *Ca*AFOR. **c**, The tungstopterin cofactor and neighbouring residues are shown as balls and sticks with oxygen, nitrogen, sulfur, phosphorus, magnesium, iron, and tungsten coloured red, blue, light yellow, light orange, green, orange, and gray blue, respectively. **d**, R.m.s.d. of the structurally characterised aldehyde oxidase subunits aligned on the *Ca*AFOR monomer.

*C*. *autoethanogenum* is a strict anaerobe with the stunning chemolithotrophic ability to grow with pure CO as its sole energy and carbon source, suggesting specific metabolic adaptation^6^. The bacterium encodes two putative AFOR isoforms, one of them being particularly abundant under CO conditions^20^. The bacterium was, therefore, cultivated on CO to ensure sufficient enzyme quantities for structural and biochemical characterization. The native anaerobic purification led to a homogeneous monomeric population with a molecular weight around 66 kDa, as determined by denaturing PAGE, gel filtration and high-resolution native PAGE (Extended Data Fig. 1). The protein identity, previously named ‘AOR1’ (ref. ^8^) (WP_013238665, expected molecular weight of 66,335 Da, here abbreviated as *Ca*AFOR), was confirmed by mass spectrometry (MS)-based proteomics (Supplementary Fig. 1). This protein is similar to the homologs from *Peptoclostridium acidaminophilum*^21^, *Thermoanaerobacter* sp. X514 (ref. ^14^) and *Pyrococcus furiosus*^22^ (sequence identity of 74.55%, 67.83% and 55.61%, respectively, for a sequence coverage of 100%, 99% and 99%), constituted by a single peptide and proposed to use ferredoxin as an electron donor and acceptor, unlike the heterohexameric NADH-dependent enzyme from *Aromatoleum aromaticum*^12^ (sequence identity 45.00%, sequence coverage 99%; Extended Data Figs. 2 and 3a). These proteins belong to a larger subgroup of proteins, phylogenetically separated from other types of aldehyde oxidases, such as the formaldehyde:ferredoxin oxidoreductase (FFOR), the benzoyl-CoA reductase BamB or the aliphatic sulfonate:ferredoxin oxidoreductase^23^ (ASOR) (Extended Data Fig. 2). All these oxidoreductases contain two metallocofactors: a [4Fe–4S] cluster and a W-containing bis-pyranopterindithiolene cofactor (simplified here as tungstopterin)^23^. Noteworthy, the monomeric organization of *Ca*AFOR is also shared in the homolog from *P*. *acidaminophilum*, while the enzymes from *Thermoanaerobacter* sp. X514 and *P*. *furiosus* organize as a homodimer^23^. This latter is stabilized in the structure of *Pf*AFOR by a modeled Fe atom at the dimeric interface, absent in the *Ca*AFOR structure^23^.

**Fig. 1:**
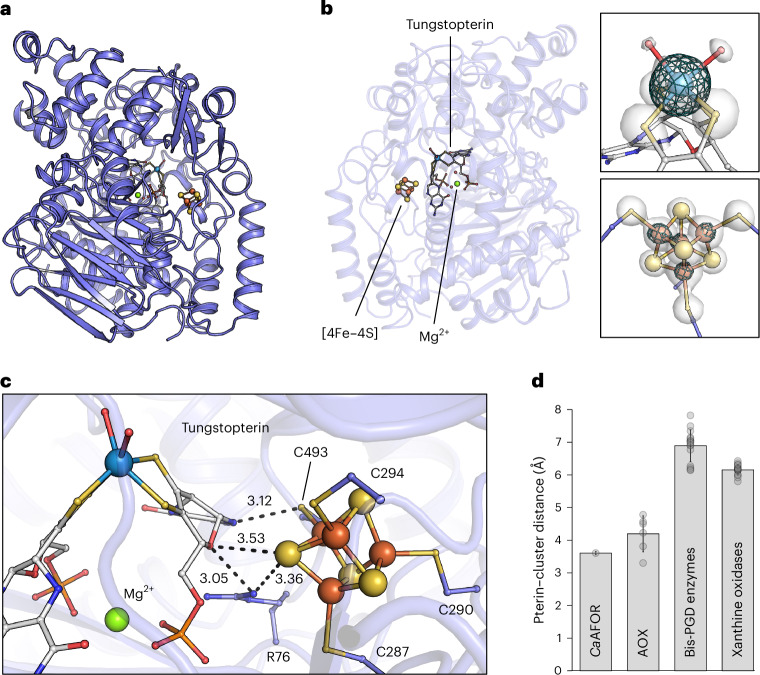
Structure of the AFOR from *C*. *autoethanogenum.* **a**, Overall structure in a cartoon model. **b**, Metallocofactor composition. Left, *Ca*AFOR is represented as a transparent cartoon model rotated by 180° along the *y* axis compared to **a**. Ligands are displayed as balls and sticks. Right, the 2*F*_o_ − *F*_c_ (3.5σ) and anomalous (7.0σ) maps around the cofactors are shown as a white transparent surface and a cyan mesh, respectively. **c**, Distances between the [4Fe–4S] cluster and the tungstopterin in *Ca*AFOR are represented in black dash lines and given in Å. **d**, Distances between the [4Fe–4S] cluster and the tungstopterin in structurally characterized pterin-dependent enzymes. AOX stands for all W-dependent aldehyde oxidases except *Ca*AFOR (Methods). The average and s.d. are shown, with individual data shown as transparent gray dots. The number of values used depends on the number of available structures deposited: *Ca*AFOR, one structure for two chains; AOX, six structures; bis-PGD enzymes, 16 structures; xanthine oxidases, 16 structures. For AOX, bis-PGD enzymes and xanthine oxidases, independent data points display an average of all chains in each structure. In **a**–**c,** *Ca*AFOR is shown as a slate blue cartoon with the cofactors and coordinating residues shown as balls and sticks with carbon, oxygen, nitrogen, sulfur, phosphorus, magnesium, iron and tungsten colored white (slate for residues), red, blue, light yellow, light orange, green, orange and gray blue, respectively. Source data

**Fig. 2:**
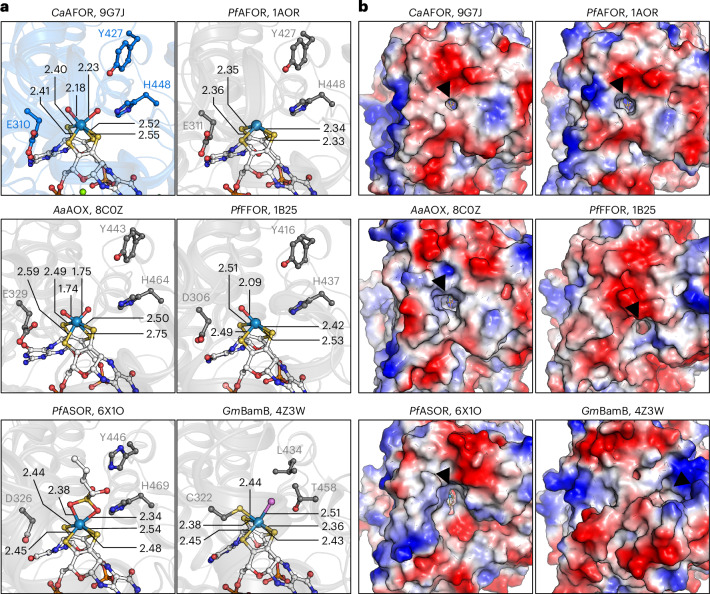
Active site and its entrance across structural homologs. **a**, W coordination in *Ca*AFOR and structurally characterized homologs. Proteins are shown in transparent cartoons colored slate (*Ca*AFOR) or gray. Cofactors, coordinating residues and ligands are shown as balls and sticks with carbon, oxygen, nitrogen, sulfur, phosphorus, magnesium and tungsten colored white (or according to the protein for residues), red, blue, light yellow, light orange, green and gray blue, respectively. The unidentified atom in the *Gm*BamB structure (PDB 4Z3W) is colored light purple. Distances between the W and binding atoms are indicated in Å. **b**, Surface and access to the active site. Proteins are shown as surface colored by charge distribution from negative (red) to positive (blue). The opening connecting the active site to the solvent is indicated with a black arrow.

The as-isolated *Ca*AFOR did not show any activity when monitoring acetaldehyde oxidation with either methyl viologen (MV, respectively MV^2+^/MV^+●^ for oxidized and reduced state) or benzyl viologen (BV, respectively BV^2+^/BV^+●^), following the classic assay used by the community. This inactivation phenomenon is known for homologous enzymes^24–26^ and was an additional motivation to solve the *Ca*AFOR structure. X-ray fluorescence measurement on protein crystals confirmed the presence of W in *Ca*AFOR (Supplementary Fig. 2) and the structure was experimentally solved by single-wavelength anomalous diffraction at the W L3 edge. The final model was refined to 1.59-Å resolution (Protein Data Bank (PDB) 9G7J; Extended Data Table 1), the best resolution obtained for this group of enzymes, granting high confidence to model the metallocofactors (Fig. 1). The tungstopterin cofactor of *Ca*AFOR exhibits a canonical composition with two pyranopterindithiolene groups, stabilized in a ‘bent conformation’ by the coordination of the flanking phosphates with a Mg atom (Supplementary Fig. 3). The crystal asymmetric unit contains two *Ca*AFORs, which have too weak of an interaction to be stable in solution, confirming the monomeric state, unlike structurally characterized homologs (Extended Data Fig. 3a). However, the overall monomeric structure is highly similar to the structures of the homologs, the AFOR from *P. furiosus* being the closest (Extended Data Fig. 3b–d). While some positions are perfectly conserved in the active site, which would be candidates to support catalysis, some discrepancies are seen, probably because of fine-tuning of substrate specificities across the different enzymes (Fig. 2a, Extended Data Fig. 3c and Supplementary Fig. 4). The active site, embedded in a rather hydrophobic cavity, connects to the solvent by narrow hydrophobic tunnels, similar to other aldehyde oxidases (Fig. 2b and Supplementary Fig. 4b). As in homologs, a short distance separates the two cofactors (Fig. 1c,d), allowing efficient electron transfer.

The W atom, confirmed by its anomalous signal (Fig. 1b), shows no proteinogenic covalent ligand as all other structurally characterized homologs except the benzoyl-CoA reductase BamB (Fig. 2a). The W atom is coordinated by the dithiolene groups from each pterin and by two atoms modeled as oxygen according to the electron density (Fig. 1b). A similar ligand has been modeled in the structure of the aldehyde oxidoreductases from *A*. *aromaticum* (PDB 8C0Z)^27^ based on the reanalyzed diffraction data from the *P*. *furiosus* enzyme and the extended X-ray absorption fine structure (EXAFS) spectroscopy data previously obtained on the same enzyme^28^. The W–O distances in the *Ca*AFOR structure are, however, substantially longer (around 2.2 Å) than the W=O double bond modeled in the structure from *A*. *aromaticum* (1.74–1.75 Å) or detected by EXAFS spectroscopy (1.74 Å)^28^. However, it is similar to the W–OH bonds calculated by QM/MM (2.06 Å)^27^, also proposed by EXAFS spectroscopy (W–O bond of 2.16 Å)^28^ and slightly shorter than the W­–O­ bonds modeled in the ASOR of *P*. *furiosus* (2.34–2.38 Å, PDB 6X1O)^29^ (Fig. 2a). On the basis of the observed distance, the ligands were modeled as hydroxo rather than oxo groups (Figs. 1b and 2a).

### Ferredoxin is a critical component of *Ca*AFOR activity

**Fig. 3:**
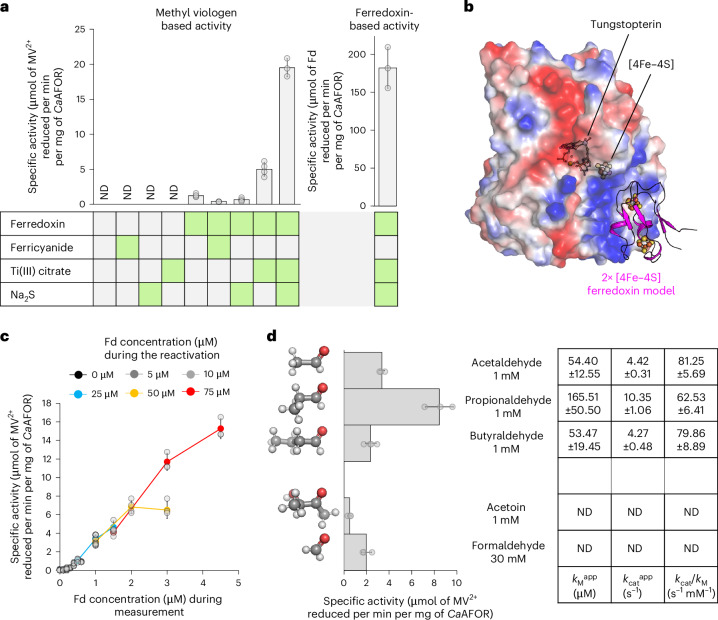
Ferredoxin-dependent AFOR activity. **a**, Left, MV^2+^-based *Ca*AFOR activity after incubation with 250 µM ferredoxin, 500 µM ferricyanide, 500 µM Ti(III) citrate and/or 500 µM sulfide (Na_2_S). Right, *Ca*AFOR activity with ferredoxin as electron acceptor (after reactivation). Green boxes highlight when the ferredoxin and chemicals are present. **b**, AlphaFold2 model of *Ca*AFOR with the ferredoxin. *Ca*AFOR is displayed as a transparent surface colored by charge distribution from negative (red) to positive (blue). The ferredoxin is presented as a pink cartoon. Metallocofactors are shown in balls and sticks and colored as in Fig. 1. The model is deposited on Zenodo^54^. **c**, Impact of the ferredoxin concentration on activity measurements. The enzyme was incubated (interpreted here as a ‘reactivation phase’) with different concentrations of ferredoxin represented by different colors. Three different final concentrations of ferredoxin were assessed during activity measurements for each reactivation procedure. MV^2+^ served as an electron acceptor. **d**, Aldehyde:MV^2+^ oxidoreductase activity (expressed in µmol of reduced viologen per min per mg of enzyme) of the *Ca*AFOR with different aldehydes (shown as balls and sticks with carbon, oxygen and hydrogen colored as gray, red and white). The final concentrations of aldehydes used for activity measurements are indicated next to the graph. The kinetic parameters with the different aldehydes are indicated in the table. In **a**,**d**, ND denotes not detectable. In **a**,**c**,**d**, the average and s.d. are shown, with individual data shown as transparent gray dots. The measurements were performed in replicates (*n* = 3 or 4) from independent treatments of different reactivations of the same enzyme pool. Source data

The *Ca*AFOR specific activity in the cell extract exhibited a progressive decrease upon dilution (Supplementary Fig. 5). This led us to hypothesize four scenarios: (1) the physiological molecular crowding in the cell (for example, high protein concentration) might be important to maintain *Ca*AFOR integrity; (2) the redox potential of the solution is critical to maintaining the metallocofactor in a catalytical competent state; (3) the tungstopterin is losing a hypothetic sulfidoligand necessary for its activity; and (4) a molecule present in the extract is required to stabilize the *Ca*AFOR active state. The four hypotheses were tested as follows. Firstly, as a standard procedure, bovine serum albumin (BSA,which does not undergo interactions with other proteins) was added to maintain a high protein concentration and mimic molecular crowding during dilution of the cell extract. However, its addition did not impact the specific activity (Supplementary Fig. 5). Secondly, to test whether the redox state of the solution could influence the metallocofactor reactivity, we either used a reducing agent (Ti(III) citrate) or a mild oxidant (ferricyanide) on the pure enzyme. The incubation with either redox agent was inefficient in detecting enzymatic activity (Fig. 3a). Thirdly, sulfide was previously shown to reactivate certain AFOR homologs^24^. Supplementing sulfide (as Na_2_S) might lead to the spontaneous incorporation of a sulfidoligand on the tungstopterin. This hypothetical axial ligand common to many molybdopterin and tungstopterin enzymes might be essential for catalysis. Nevertheless, its addition did not lead to any detectable activity of the pure *Ca*AFOR (Fig. 3a). Lastly, a molecule still present in the cell extract might be necessary to promote an active state of the enzyme through an unknown mechanism. The small electron-carrying protein ferredoxin, the supposed physiological electron acceptor of the *Ca*AFOR, could have such a role. Hence, we investigated whether the native ferredoxin from *C*. *autoethanogenum*, one of the most prominent proteins in the bacterium, could be used to restore the enzyme activity by monitoring aldehyde oxidation.

**Extended Data Fig. 4:**
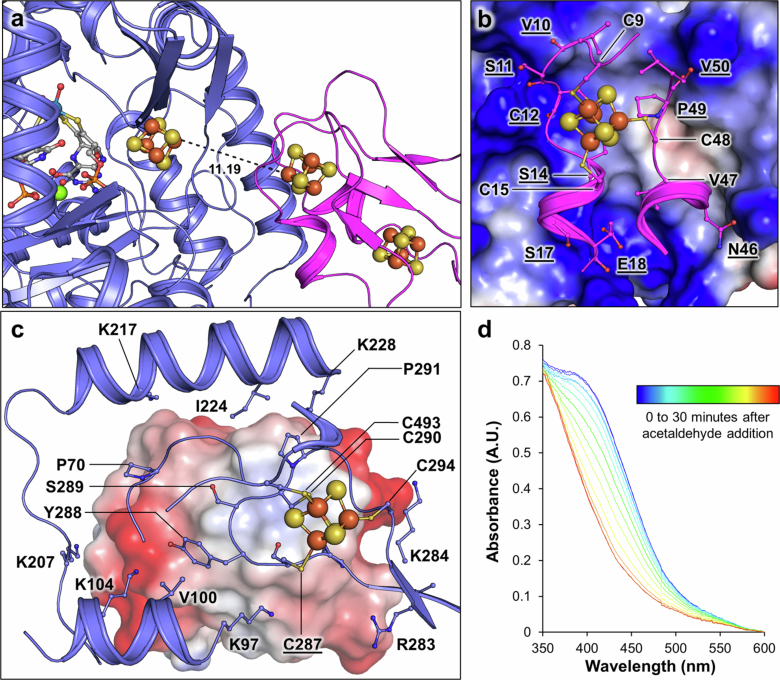
Ferredoxin docking and reduction by *Ca*AFOR. **a**, Distances (indicated in Å) between ferredoxin and *Ca*AFOR clusters in the AlphaFold2 model presented in Fig. 3b. **b, c**, Mapping of the *Ca*AFOR-ferredoxin interface. The ferredoxin docking on *Ca*AFOR (with *Ca*AFOR shown as surface coloured by electrostatic charges) and the *Ca*AFOR docking on ferredoxin in a different orientation (with the ferredoxin shown as surface coloured by electrostatic charges) are shown in **b** and **c**, respectively. **a – c**, Proteins are shown as cartoons, and *Ca*AFOR and ferredoxin are coloured slate and pink, respectively. Residues involved in [4Fe-4S] cluster coordination and docking stabilisation are shown as balls and sticks. Residues interacting (hydrogen bond and Van der Waals contacts) with main chains (with or without the side chain) are underlined. Cofactors are shown as balls and sticks, with carbon, oxygen, nitrogen, sulfur, phosphorus, magnesium, iron, and tungsten coloured white (as the respective proteins for residues), red, blue, light yellow, light orange, green, orange, and gray blue, respectively. **d**, Ferredoxin reduction by *Ca*AFOR, monitored by measuring the absorbance spectrum every 2.25 min. Spectra are coloured blue to red from the first to the last time point. Source data

With a final purification yield of 614 ± 131 nmol of ferredoxin per gram of soluble proteins obtained after lysis (average and s.d. obtained from three independent purifications), the protein is one of the most abundant from *C*. *autoethanogenum*. This is coherent with previous transcriptomics analyses^19,30^, showing that the gene encoding the ferredoxin is among the most expressed in the organism. As a comparison, the CODH/ACS^6^, the key catabolic enzyme of the bacterium^6,20^, is around tenfold lower in terms of purification yield (55 ± 28 nmol g^−1^ of soluble extract, obtained from five independent purifications), and 2–20-fold lower in transcriptomic analysis^19,30^. MS-based proteomics (peptide identified as WP_013236834, OVY50687.1; Supplementary Fig. 1) did not suggest any other ferredoxin present in the purified preparation. Therefore, the protein is most probably the central physiological electron donor and acceptor of the ferredoxin-dependent reactions occurring in the bacterium and is similar to the characterized homolog from *Clostridium* *pasteurianum* (WP_003440320, 78.57% sequence identity) used in many studies as an electron acceptor and donor for oxidoreductases^4,5,31,32^. An AlphaFold2 prediction of the *Ca*AFOR–ferredoxin interaction displays a docking of the ferredoxin on a positively charged patch near the [4Fe–4S] cluster of the *Ca*AFOR at a distance (11.2 Å) short enough for fast electron transfer (Fig. 3b and Extended Data Fig. 4a–c). The charge distribution of the putative docking area is reminiscent of that recently described in the CODH–ferredoxin complex^11^. Accordingly, the ferredoxin (natively isolated in its oxidized state, Supplementary Fig. 6) was used as an electron acceptor and was reduced by the purified *Ca*AFOR upon acetaldehyde addition (Extended Data Fig. 4d).

**Extended Data Fig. 5:**
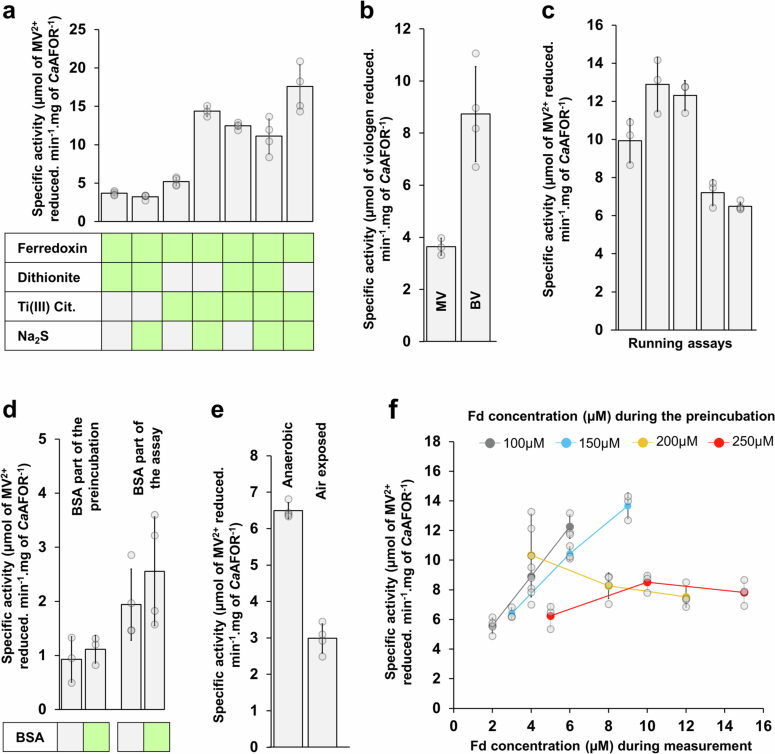
Enzyme reactivation and activity. **a**, Reactivation of *Ca*AFOR with 250 µM ferredoxin, 500 µM dithionite, 500 µM Ti(III) citrate and/or 500 µM sulfide. Experiment 7 has been performed under the same conditions as Experiment 4 on the same day. **b**, Activity of *Ca*AFOR with MV^2+^ and BV^2+^ (both at 5 mM final) as electron acceptors. **c**, Activity measurements in multiple reactivations of the same pool of frozen enzyme. **d**, Effect of BSA during reactivation (left) or activity measurements (right). **e**, *Ca*AFOR overnight incubation under anaerobic or aerobic conditions followed by reactivation and activity measurements. Reactivation procedure and activity measurements were performed in anaerobic conditions for both. **f**, Impact of the ferredoxin concentration during reactivation and activity measurements. The enzyme was reactivated with different concentrations of ferredoxin (represented by different colours). By dilution, three different final concentrations of ferredoxin were assessed during activity measurements for each reactivation procedure. **a** and **c-f**, MV^2+^ serves as an electron acceptor. **a-f**, Average and standard deviation are shown, with individual data shown as transparent gray dots. The measurements were done in replicates (n = 3 or 4), from independent treatments of different dilutions of the same enzyme pool. Source data

A preincubation of *Ca*AFOR with ferredoxin led to a detectable MV^2+^-dependent acetaldehyde oxidation activity (Fig. 3a). The preincubation effect was enhanced by the presence of a reducing agent such as Ti(III) citrate and, more efficiently, when combined with sulfide (up to tenfold; Fig. 3a). Using dithionite instead of Ti(III) citrate also enhanced the activity but the effect was not affected by sulfide addition, probably because the dithionite solution already contained sulfide (Extended Data Fig. 5a and Supplementary Fig. 7). Replacing MV^2+^ by BV^2+^ increased activity around 2.4-fold, which was expected considering the reaction thermodynamics (*E*^0^′ = −374 mV for BV instead of *E*^0^′ = −450 mV for MV^33^; Extended Data Fig. 5b). In addition to being reproducible, the optimized protocol of preincubation with ferredoxin, Ti(III) citrate and sulfide presented fluctuations of the specific activity that could not be corrected by addition of BSA (Extended Data Fig. 5c,d).

Because adding reducing agents strongly stimulated *Ca*AFOR activity during the preincubation phase, we speculated that the as-isolated enzyme is in an inactive oxidized state. Accordingly, incubation of the as-isolated *Ca*AFOR with BV^2+^ indicated a globally fully oxidized state of the purified enzyme (10.29 ± 3.35 µM of transferable electrons from 102.46 µM *Ca*AFOR; Supplementary Fig. 8). This suggests a spontaneous oxidation occurring after cell disruption under anaerobic conditions, similar to other metalloenzymes^34^ and echoing previous observation on AFOR homologs losing activity over purification^24–26^. A reactivation process performed on overnight exposition of *Ca*AFOR with air yielded 45% of the activity compared to an enzyme stored in an anoxic environment (Extended Data Fig. 5e), a decrease that might be due to the loss of the [4Fe–4S] cluster. While all experiments indicate that enzyme oxidation is reversible, the impact of the preincubation phase on the enzyme is more complex to interpret and might hide a more intricate reactivation process in which ferredoxin association has a key role.

To decipher the effect of ferredoxin on *Ca*AFOR activity, we measured the ferredoxin reduction rates instead of using viologen as an electron acceptor. After enzyme reactivation, we measured a specific activity of 182.12 ± 26.97 µmol of reduced ferredoxin (assuming a single electron transferred to ferredoxin) per min per mg of *Ca*AFOR (Fig. 3a), a high activity for this type of enzyme^12,14,21,25,35–39^ (expressed in µmol of aldehyde oxidized per min per mg of enzymes in certain of the cited references). The tenfold difference observed between the reduction rates of MV^2+^ and ferredoxin contradicts the possibility that ferredoxin only serves as an electron bridge between AFOR and MV^2+^. It rather strengthens the hypothesis of a role that might indeed contribute to *Ca*AFOR reactivation by stimulating the reduction of the tungstopterin. To deepen our investigation, different concentrations of ferredoxin were tested during both reactivation and activity measurements. Up to 75 µM, the activity increases linearly with the concentration of ferredoxin used during reactivation (Fig. 3c). Higher ferredoxin concentrations did not enhance enzyme reactivation (Extended Data Fig. 5f). The ferredoxin concentration during activity measurement also positively influences the measured activity, this later increasing with the ferredoxin concentration up to 5–10 µM ferredoxin during activity measurement. A large excess of ferredoxin is, hence, necessary for a maximal reactivation of the enzyme but a relatively high ferredoxin concentration is also important to maintain *Ca*AFOR activity. This echoes with the large excess of ferredoxin necessary for maximal activity of the enzyme of *Thermoanaerobacter* sp. X514 and explains the loss of *Ca*AFOR activity in the extract upon dilution, as it decreases the concentration of native ferredoxin.

### *Ca*AFOR substrate specificity

The affinity of *Ca*AFOR for different aldehydes was tested. Because of the observed fluctuations during the reactivation procedure (Extended Data Fig. 5c), the estimated apparent kinetic parameters for each substrate exhibited relatively large deviations. The results indicated that *Ca*AFOR catalyzes the oxidation of acetaldehyde, propionaldehyde and butyraldehyde with a turnover and affinity in the same order of magnitude (Fig. 3d). The enzyme turnover and *k*_M_^app^ with these substrates are in the range that is determined in characterized homologs^12,14,25,26,37,39^. This relative nonspecificity is a common feature of the enzyme family^14,21,35,39^. Nevertheless, *Ca*AFOR poorly catalyzed formaldehyde oxidation, requiring concentrations above the confidence measurements to extract kinetic parameters. Using branched aldehydes such as acetoin as substrate only yielded extremely low activity. The enzyme selectivity could be due to both the characteristics of the active site and the hydrophobic channel connecting this latter to the solvent (Fig. 2b). More insights were gained on the substrate diffusion to the active site by detecting the cavities connecting the tungstopterin to the solvent and analyzing molecule diffusion (Supplementary Fig. 9). While the prime access shown in Supplementary Fig. 4b (tunnel 1) present an optimal track, a second narrower tunnel was also detected with two major bottlenecks limiting appropriate diffusions. Ligands docking on the rigid *Ca*AFOR structure showed that the binding energy of acetaldehyde and larger aldehydes in the tunnel is more negative than that of formaldehyde, suggesting a less efficient diffusion of the small aldehyde. The binding energies of bulkier substrates, less efficiently oxidized by *Ca*AFOR (that is, butyraldehyde and acetoin), are more negative than those of acetaldehyde and propionaldehyde. This indicates that ligand docking alone cannot explain the observed enzyme selectivity. Other events are probably involved in the observed difference in kinetic parameters, such as the optimal positioning of the aldehyde group in front of the tungstopterin in a catalytically competent conformation. Notably, the docking of acetic acid would be slightly more efficient than that of acetate, although without a dramatic difference.

The homolog from *A*. *aromaticum* was recently shown to exhibit hydrogenase activity^38^. In our setup, we could not detect H_2_ oxidation activity by *Ca*AFOR with either MV^2+^ or ferredoxin as an electron acceptor (with tested H_2_ final concentrations of 0.96%, 2.75%, 4.5% and 29% in the gas phase). Unlike the homolog from *A*. *aromaticum*^38^, H_2_ does not exhibit any inhibitory effect on the aldehyde:MV^2+^ oxidoreductase activity of *Ca*AFOR (Supplementary Fig. 10). In *C*. *autoethanogenum*, H_2_ oxidation is rather operated by dedicated hydrogenases that have been suggested to catalyze H_2_ formation during the CO-dependent growth^4^.

### CO-driven alcohol production

**Fig. 4:**
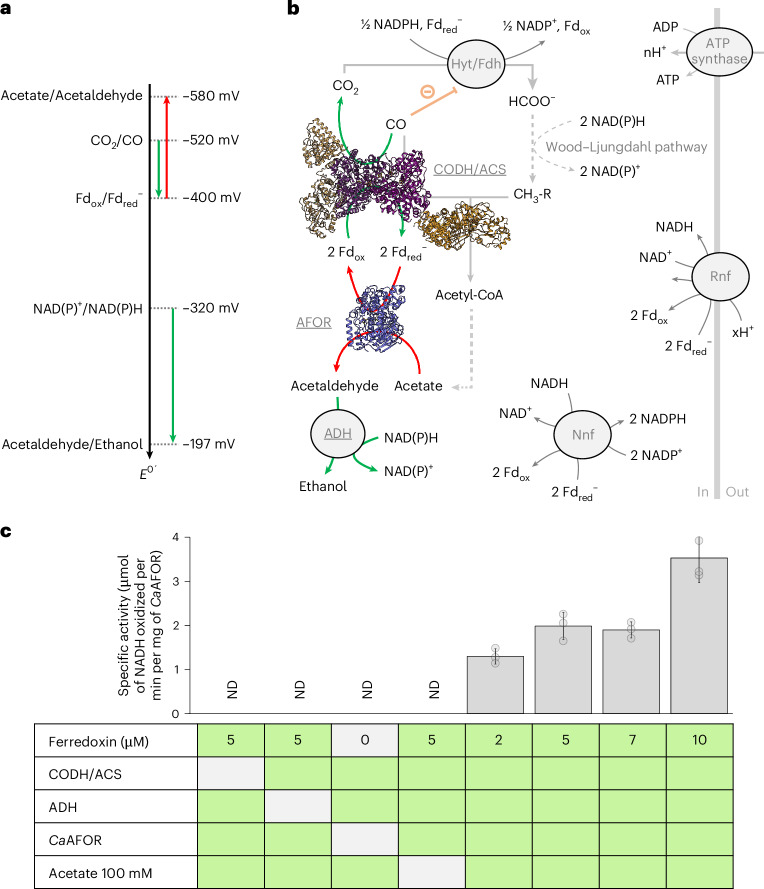
Coupled enzymatic assay for in vitro CO-dependent ethanol production. **a**, Standard redox potential of the redox couples involved in ethanol production. Thermodynamically favorable and unfavorable reactions are shown by green and red arrows, respectively. **b**, Metabolic pathway for ethanol production in *C*. *autoethanogenum*. The experimental *Ca*CODH/ACS and *Ca*AFOR structures are shown in cartoon representation^6^. CO inhibition affecting the electron-confurcating Hyt/Fdh complex is colored orange. Dotted lines indicate multistep reactions and CoA is omitted for clarity. Enzymatic reactions used in the coupled assay are colored green and red, as in **a** (ADH from *S*. *cerevisiae* is used instead of *Ca*ADH). The numbers of protons translocated through ferredoxin oxidation and for ATP synthesis was not experimentally determined and, therefore, are denoted as x and n, respectively. **c**, Specific activity in the different conditions for the coupled assay. The average and s.d. are shown, with individual data displayed as transparent gray dots. The measurements were performed in replicates (*n* = 3), from independent treatments of different reactivations of the same enzyme pool. Green boxes contain the proteins and acetate. Source data

Following the most common scenario in *C*. *autoethanogenum*, *Ca*AFOR should be the starting point of the ethanol pathway by generating acetaldehyde from acetate. As expected, no reliable in vitro acetate reduction by *Ca*AFOR could be monitored with MV^+●^, BV^+●^ or reduced ferredoxin because of the unfavorable thermodynamics of the reaction (Fig. 4a). The reaction equilibrium is probably reached before a reliable activity could be monitored. In vivo, the physiological context is drastically different as the favorable acetaldehyde reduction to ethanol coupled to NAD(P)H oxidation would prevent the aldehyde accumulation, while the ferredoxin pool would be maintained in a reduced state by CO oxidation (Fig. 4a,b). Therefore, the overall process from acetate to ethanol would be largely exergonic. To mimic this metabolic thermodynamic coupling with exergonic reactions, *Ca*AFOR was coupled to the NADH-dependent ADH from *Saccharomyces* *cerevisiae* and the native CODH/ACS complex^6^ to fuel the acetate-reducing reaction with electrons from CO oxidation (Fig. 4b). The experiment was performed sequentially by testing the compatibility of the three different functional modules (electron donation, acetate reduction and acetaldehyde elimination).

**Extended Data Fig. 6:**
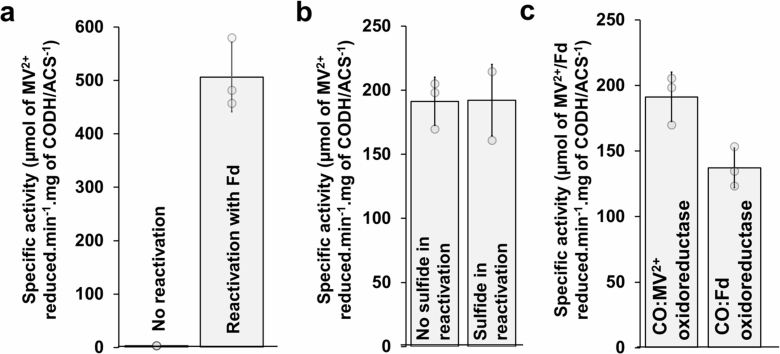
Reactivation and activity of the *Ca*CODH/ACS. **a**, Specific activity of the CODH/ACS^6^ without and with reactivation by incubation with ferredoxin and Ti(III) citrate. **b**, Impact of sulfide (500 µM final) on the CODH/ACS reactivation process. **c**, CO:MV^2+^ and CO:ferredoxin oxidoreductase activity of the CODH/ACS complex^6^ after reactivation. **a-c**, Average and standard deviation are shown, with individual data shown as transparent gray dots. The measurements were done in replicates (n = 3), from independent treatments of different dilutions of the same enzyme pool. Source data

We previously noticed that the CODH/ACS complex from *C*. *autoethanogenum* drastically loses activity during purification^6^. When the reactivation procedure developed for the *Ca*AFOR was applied to the purified CODH, a 150-fold increase in MV^2+^-based CO oxidation activity was measured (Extended Data Fig. 6a). Loss of activity over time was, however, still observable despite the enzyme reactivation. Unlike the *Ca*AFOR, the reactivation of CODH activity is not impacted by sulfide addition and the activity with MV^2+^ as an electron acceptor is around 40% higher than that measured with ferredoxin (Extended Data Fig. 6b,c).

**Extended Data Fig. 7:**
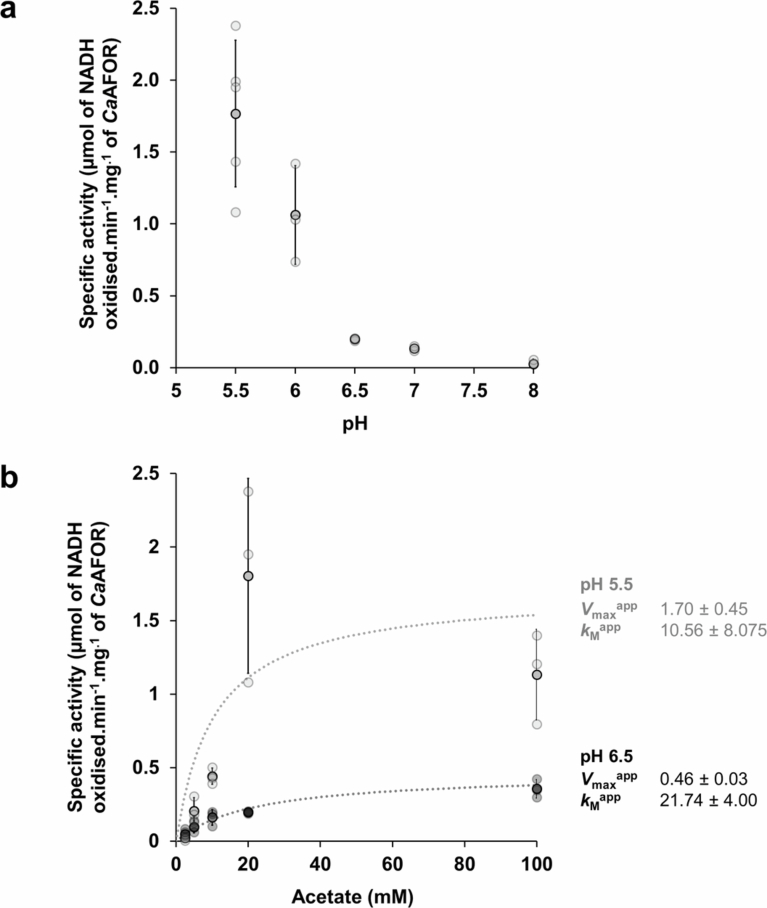
pH and concentration impact on the monitored coupled assay. **a**, **b**, Specific activity (per mg of *Ca*AFOR) in the coupled assay depending on pH (**a**) and sodium acetate concentration (**b**). **b**, Measurements were performed at pH 5.5 (light gray) or 6.5 (dark gray). With a p*K*_a_ of 4.76 for acetic acid/acetate, the estimated *k*_M_^app^ of *Ca*AFOR for acetic acid would represent around 1.63 and 1.18 mM in the solution at pH 5.5 and 6.5, respectively. **a, b**, Average and standard deviation are shown, with individual data shown as transparent gray dots. The measurements were done in replicates (n = 3 or 5), from independent treatments of different dilutions of the same enzyme pool. Source data

The CO-driven ethanol synthesis was performed with CODH/ACS and ADH in saturating concentration, such that *Ca*AFOR would be the rate-limiting enzyme in the process. Firstly, we confirmed that AFOR and NADH:acetaldehyde oxidoreductase activities were not impacted by the addition of CO (104.6% ± 10.0% and 92.5% ± 18.0% of the activity with around 29% CO in the gas phase, respectively). Secondly, we confirmed the absence of a NADH oxidase background when all components were added except acetate. The oxidation of NADH reached a robust specific activity when all components were added to the coupled assay, indicating that the *Ca*AFOR produces acetaldehyde under this condition (Fig. 4c). The NADH oxidation rate was dependent on CO concentration, as around 3% final CO in the gas phase was not sufficient to detect reliable activity and was impacted by ferredoxin concentration (Fig. 4c). It cannot be deciphered whether the effect of ferredoxin concentration is because of *Ca*AFOR or CODH reactivation and/or electron transfer from CODH/ACS to *Ca*AFOR. The kinetic parameters for acetate were affected by the pH, with the enzyme exhibiting higher rates at acidic pH (Extended Data Fig. 7). The maximal monitored acetate reduction turnover by *Ca*AFOR at 9.20 ± 0.74 s^−1^ (obtained under the same conditions than Fig. 4c, with 10 µM ferredoxin) is on the same order of magnitude as the rates obtained during the thermodynamically favorable aldehyde oxidation with viologen derivatives catalyzed by *Ca*AFOR or homologs with similar organization^14,21^ (spanning a turnover range from 5 to 50 s^−1^) and around 1,000-fold higher than the acid reduction turnover determined with the closely related enzyme from *Thermoanaerobacter* sp. X514 (turnover of 0.015 s^−1^)^14^.

## Discussion

The carbon-cycling economy is one of the most important challenges that our modern society faces to counterbalance greenhouse gas emissions and ensure long-term sustainability. An existing solution is to rely on the chemistry of acetogenic bacteria to convert industrial greenhouse waste gases (for example, syngas released from the steel mill industry) into alcohols used as biofuels or as building blocks for organic chemistry. While *C*. *autoethanogenum* is in the spotlight of this technology, its highly specialized CO metabolism remains to be unveiled at the molecular level. While we previously characterized the CO-capturing strategy of the CODH/ACS complex^6,11^, we describe in this work the acetate reduction step catalyzed by a W-dependent AFOR for ethanol production.

By performing the reversible conversion of different aldehydes into acids, AFORs are environmentally and biotechnologically relevant enzymes, putatively valuable for synthetic biology^38,40^. Numerous mechanisms have been proposed on the basis of structural and biophysical characterization or molecular simulations^23,27,29,41,42^. In all of them, the conserved glutamate and histidine (Fig. 2a, Extended Data Fig. 3c and Supplementary Fig. 4a) would assist the reaction for proton exchange, while the reaction intermediates and the role of the tungsten hydroxo and oxo ligands differ. The distance separating the catalytic W atom and the axial ligands better fits with hydroxyl groups in the near-atomic resolution structure of the as-isolated *Ca*AFOR. One of the hydroxyl groups establishes short hydrogen bonds with the perfectly conserved glutamate (E312) and histidine (H448) (Supplementary Fig. 4a), a position that should be occupied by the acetaldehyde and acetate molecules and reaction intermediates. During the reactivation and under catalytic turnover, the W would undergo different redox states, disrupting these axial ligands and leaving a vacant position to bind the substrate. To evaluate the *Ca*AFOR redox states observed in the crystal structure, we incubated the as-isolated protein with an excess of BV^2+^ and barely detected a reduction of the artificial electron acceptor (with an estimated 0.1 mol of electrons per mol of *Ca*AFOR; Supplementary Fig. 8). This suggests that *Ca*AFOR was crystallized mostly in a fully oxidized W(VI) state. However, the cofactor may be in an inactive state different from the catalytically competent W(VI) occurring under turnover conditions, as the as-isolated enzyme requires strong reducing agents to restore its catalytic potential. Moreover, it is worth noting that the W coordination observed in the crystal structure might represent a mixture of different states because of X-ray radiation damage, known to cause aberrant reduction that limits the interpretation of its state. A similar putative alteration of the active site is also possible in the previous structural investigation on AFOR homologs (by X-ray diffraction and electron microscopy). Therefore, further studies are crucial to better understand the W chemistry in these enzymes.

**Extended Data Fig. 8:**
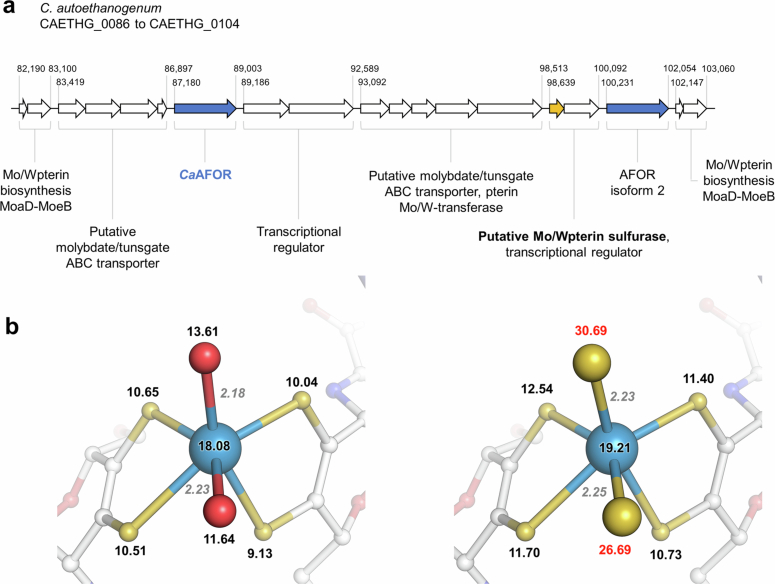
Genomic environment and sulfur ligand in *Ca*AFOR. **a**, Genomic environment of the genes coding for the *Ca*AFOR and the second AFOR isoform of *C. autoethanogenum* (slate). Genes are shown as arrows with lengths depending on the gene length. Adjoining arrows represent operonic structures predicted by the Operon Mapper webserver^68^. The gap between predicted operons is not representative of the actual sequence length. The first and last positions of the predicted operons are indicated, together with the predicted function of the encoded proteins. The putative W/Mo-pterin cofactor sulfurase is coloured orange. **b**, Distances and b-factors of the W atom and coordinating ligands where S atoms substitute the O ligands. The cofactor and ligands are shown as balls and sticks with carbon, oxygen, nitrogen, sulfur, and tungsten coloured white, red, blue, light yellow, and gray-blue, respectively. B-factors are coloured black and distances between the W and axial O/S ligands in gray (given in Å). After refinement, the modelled S atoms have a b-factor (labeled in red) that is inconsistent with the surrounding atoms. As reactivation of the enzyme can be performed without sulfur sources, the putative S-ligand atom in the active site could not be necessary for activity, similar to the sulfido ligand of the Mo-dependent trimethylamine-N-oxide reductase^46^. The absence of the S-axial ligand prone to oxidation in some enzymes may explain the relative tolerance to oxygen of aldehyde oxidases of facultative aerobic bacteria^12^. The higher electronegativity and charge density of sulfur compared to oxygen could be more favourable in the reaction, explaining why sulfide enhances enzyme activity without being necessary.

Compared to other oxidoreductases that can be reactivated with only reducing agents (for example, certain CODHs^43^ or hydrogenases^44^), *Ca*CODH and *Ca*AFOR additionally require ferredoxin. We suspect that ferredoxin docking would provide an electron path from Ti(III) citrate to the metallocenter through the [4Fe–4S] cluster without provoking large conformational rearrangements because of the robustness of the protein scaffold. One key difference between the two enzymes is the stimulation of sulfide in the case of *Ca*AFOR. A ferredoxin-independent reactivation by sulfide was previously described for the FFOR from *P*. *furiosus*^24^ and dithionite (a sulfide source) is regularly used in the purification of AORs to preserve the activity^24,25^. The genomic environment of the gene encoding *Ca*AFOR contains a gene coding for a putative molybdopterin and tungstopterin cofactor sulfurase, homologous to the gene responsible for sulfur addition on the Mo atom in xanthine oxidases^45^ (Extended Data Fig. 8a). Hence, in its physiological state, this AFOR might present at least one sulfur-based ligand (sulfhydryl or sulfido) on the tungsten, similar to the protein belonging to the DMSO reductase family^46^ (for example, formate dehydrogenases^47^) and Mo-dependent aldehyde oxidase^48^. The progressive oxidation of the ligand during the purification would explain the gradual inactivation previously observed^22,24,25,49^ and the presence of oxygen rather than sulfur in the presented structure (Extended Data Fig. 8b). Despite our efforts to soak or cocrystallize the enzyme with ferredoxin, dithionite and/or sulfide, no notable modification of the electron density could be observed in the crystal structure, an effect most probably due to the crystal packing preventing interaction with the ferredoxin in crystallo. Neither the previously published structural data by crystallography or cryo-electron microscopy nor the EXAFS spectroscopy data suggested a sulfur atom as an axial ligand of the W atom in the AFOR group (the cysteine coordination in *Gm*BamB being a notable exception); however, these results could also be representative of oxidized or altered states of the enzymes. Alternatively, the role of sulfide could be important for enzyme reactivation but not as a W axial ligand. Further investigation is required to decipher whether a sulfur atom has a role in the reactivation or reaction mechanism. Obtaining additional data on the reactivated enzyme will also shine light on the precatalytic state, which might corroborate the recent work performed on the enzyme from *A*. *aromaticum* or present an unexpected reconfiguration of the W environment.

AFORs have been shown to artificially reduce acids in vitro; however, because of the thermodynamics of the reaction, the low measured rates^38,40^ and the poor affinity of the characterized enzymes for acids, this reaction has sometimes been claimed as physiologically irrelevant^12–14^. Nevertheless, when mimicking the metabolic context by constantly overflowing the system with CO-dependent ferredoxin reduction and eliminating the product with an ADH, we showed that *Ca*AFOR catalyzes a robust aldehyde production (Fig. 4c), strengthening its role in alcohol production as previously suggested in acetogenic bacteria and as demonstrated in archaea^8–10^. Our results, hence, provide biochemical verification of the conclusions of the metabolic modeling studies in *C*. *autoethanogenum*^16–18^. The most probable explanation for the high *k*_M_^app^ of *Ca*AFOR for acetate is the thermodynamic barrier (that is, the system needs sufficient acetate quantities to push the reaction up the thermodynamic hill). The acidic pH dependency can be rationalized as follows. Firstly, at lower pH, the redox midpoint potential of the acetate–acetaldehyde couple will become more positive, making acetate reduction thermodynamically more favorable. Secondly, the stimulation of the measured rates at pH 5.5 and the hydrophobic nature of the cleft and active site would indicate that the enzyme prefers acetic acid as substrate rather than acetate (Supplementary Fig. 9), as previously proposed^5,35^. Thirdly, the low pH could improve the stabilization of a reaction intermediate such as geminal diols^27^. Fourthly, the intracellular pH of the bacterium has been estimated to be as low as 6.0 when grown in a medium with a pH of 5.3 (ref. ^5^), also facilitating acetate protonation. Lastly, the bacterial growth can tolerate concentration of acetate reaching the hundreds of millimolar range^2,3,8,50^ and it was previously proposed that internal acetate concentration might be higher than that detected in the culture medium^5^. Notably, this accumulation of acetate could be the trigger of the ethanol production, to prevent jamming of the energetic metabolism and/or acidification of the medium^51,52^. Previous studies already characterized this metabolic switch to alcohol production, allowing the consumption of reducing equivalents when the acetate concentration reaches a thermodynamic threshold, to maintain ATP homeostasis and to balance the ferredoxin, NADH and NADPH pools^16,52^. In the CO-growing cells, the *Ca*AFOR would also act as an electron exhauster, generating oxidized ferredoxin to maintain high CO oxidation rates and avoiding the accumulation of the toxic molecule in the cell that would poison acetogenesis through direct inhibition of one of its initial steps^4,53^ (Fig. 4b). This is coherent with the CO tolerance of the *Ca*AFOR and the high CO concentration required for the coupled enzyme assay. With this dual function of controlling the reduced ferredoxin pool and eliminating deleterious high acetate concentration, *Ca*AFOR possibly confers metabolic advantages under CO metabolism while providing a fantastic opportunity for bioethanol production.

## Methods

### Bacterial strains and growth conditions

*C*. *autoethanogenum* DSM 10061 was obtained from the German Collection of Microorganisms and Cell Cultures. *C*. *autoethanogenum* was cultivated under strict anaerobic conditions at 37 °C in CaGM growth medium^2^ in which Ti(III) citrate was omitted, as previously described^6^. Cells were grown chemolithotrophically under agitation (140 rpm) in 1-l Duran bottles containing 0.5 l of medium (pH 6.0) and a gas phase containing 100 kPa of 100% CO. Cell growth was monitored spectrophotometrically by measuring the optical density at 600 nm (OD_600_) and changes in headspace pressure. The cells were harvested in the late-exponential phase by centrifugation and kept frozen at −80 °C in anaerobic conditions under 100 kPa of H_2_/CO_2_ (80:20). For the purification of CODH/ACS and ferredoxin, mixotrophically grown cells were used. In these cultures, *C*. *autoethanogenum* was grown in a 10-l fermenter in 9 l of a modified CaGM medium containing only 2 g l^−1^ MES buffer and 5 g l^−1^ fructose, operating at 37 °C and 150 rpm. The fermenter was inoculated with 0.5 l of a heterotrophic culture of *C*. *autoethanogenum* (OD_600_ around 2.5) grown with 5 g l^−1^ fructose and a gas phase containing 100 kPa of H_2_/CO_2_ (80:20). After 24 h of culture under continuous bubbling with 100% N_2_, 50 ml of an anoxic solution of cysteine/HCl 4% (w/v) was added and the bubbling gas was switched to H_2_/CO_2_ (80:20). Cells were harvested after 8 h. A fermenter usually reached an OD_600_ of 2 and yielded 60 g of cells (wet weight obtained after resuspension of the cell pellet after centrifugation before the final transfer for long-term storage in a −80 °C freezer).

### Protein purification

Cell lysis and preparation of extracts were performed in an anaerobic chamber filled with an N_2_/CO_2_ atmosphere (90:10) at room temperature. The cells were lysed by three rounds of French press at around 1,000 psi (6.895 MPa). To guarantee minimum oxygen contamination, the French press cell was previously flushed with N_2_ and washed twice with anoxic buffer. Soluble extracts were prepared by ultracentrifugation at 185,500*g* for 1 h at 4 °C. Protein purification was carried out under anaerobic conditions in a Coy tent with an atmosphere of N_2_/H_2_ (97:3) at 20 °C and under yellow light. Samples were passed through a 0.2-μm filter before loading on chromatography columns. During purification, multiwavelength absorbance monitoring (at 280, 415 and 550 nm) and SDS–PAGE were used to follow the enzymes.

The purification of the native *Ca*AFOR and ferredoxin was originally performed on the basis of their color, their expected size on SDS–PAGE and the natural abundance of the proteins. They were purified several times at 20 °C under strict exclusion of oxygen and under yellow light with a similar, reproducible protocol. About 15 g (wet weight) of frozen cells were thawed and diluted with 15 ml of 50 mM Tris-HCl pH 8.0 and 2 mM dithiothreitol (DTT) before lysis. After lysis, the soluble extract was diluted with 125 ml of the same buffer. The sample was loaded on a HiTrap DEAE Sepharose FF column (3 × 5 ml; GE Healthcare) equilibrated with the same buffer. After washing with five column volumes (CVs), proteins were eluted with a 0 to 0.4 M NaCl linear gradient for 13 CVs at a flow rate of 2 ml min^−1^. The *Ca*AFOR eluted between 0.16 M and 0.19 M NaCl. Two volumes of 50 mM Tris-HCl pH 8.0 and 2 mM DTT were added to the pooled fractions before loading on a 5-ml HiTrap Q-Sepharose high-performance column (GE Healthcare) column equilibrated with 25 mM Tris-HCl pH 7.6 and 2 mM DTT. After washing with five CVs with the same buffer, proteins were eluted with a linear gradient of 0.05 to 0.45 M NaCl for 12 CVs at a 1 ml min^−1^ flow rate, with the protein of interest eluting between 0.19 and 0.24 M NaCl. The resulting pooled fraction was diluted with one volume of 25 mM Tris-HCl pH 7.6, 2 M (NH_4_)_2_SO_4_ and 2 mM DTT and loaded on a 5-ml HiTrap Phenyl Sepharose high-performance column (GE Healthcare). The sample was eluted with a linear gradient of 1.0 to 0.4 M (NH_4_)_2_SO_4_ for eight CVs at a flow rate of 1 ml min^−1^. The protein was eluted between 0.73 and 0.48 M (NH_4_)_2_SO_4_. The resulting pooled fraction was diluted with two volumes of 25 mM Tris-HCl pH 7.6, 2 M (NH_4_)_2_SO_4_ and 2 mM DTT and loaded on a Source 15PHE 4.6/100 PE column (GE Healthcare). The sample was eluted with a linear gradient of 1.75 to 0.8 M (NH_4_)_2_SO_4_ for 23 CVs at a flow rate of 1 ml min^−1^. The protein was eluted between 1.73 and 1.56 M (NH_4_)_2_SO_4_. After fraction concentration on a 10-kDa-cutoff centrifugal concentrator (nitrocellulose; Vivaspin from Sartorius), contaminants were separated by size-exclusion chromatography on a Superdex 200 Increase 10/300 GL (GE Healthcare) in 25 mM Tris-HCl pH 7.6, 10% (v/v) glycerol and 2 mM DTT with a flow rate of 0.4 ml min^−1^. *Ca*AFOR was eluted with an elution volume of 13.40 ml. The pooled fraction was concentrated and the protein was directly used for crystallization or stored at −80 °C under anoxic conditions.

The ferredoxin eluted between 0.27 M and 0.33 M NaCl at the DEAE chromatography step. Two volumes of 50 mM Tris-HCl pH 8.0 and 2 mM DTT were added to the pooled fractions before loading on a 5-ml HiTrap Q-Sepharose high-performance column (GE Healthcare), equilibrated with 25 mM Tris-HCl pH 7.6 and 2 mM DTT. After washing with three CVs of the same buffer, proteins were eluted with a linear gradient of 0.2 to 0.5 M NaCl for eight CVs at a 1 ml min^−1^ flow rate, with the protein of interest eluting between 0.36 and 0.40 M NaCl. Then, 2.5 M (NH_4_)_2_SO_4_ was added as anoxic powder to the resulting pooled fraction and the sample was loaded on a 5-ml HiTrap Phenyl Sepharose high-performance column (GE Healthcare). The sample was eluted at 1 M (NH_4_)_2_SO_4_ at a flow rate of 1 ml min^−1^. The protein was concentrated on a 3-kDa cutoff centrifugal concentrator (nitrocellulose; Vivaspin from Sartorius) and the buffer was exchanged for 25 mM Tris-HCl pH 7.6, 10% (v/v) glycerol and 2 mM DTT. The protein concentration could not be estimated using the Bradford method and was estimated by the absorbance at 390 nm of the aerobically oxidized protein using an extinction coefficient^55^ of 30,000 M^−1^ cm^−1^. The protein was directly used or stored at −80 °C under anoxic conditions.

The CODH/ACS purification protocol was previously published^6^.

### MS analysis

Proteins were in-gel digested with trypsin (Promega). The resulting peptide mixtures were analyzed by liquid chromatography (LC)–MS/MS on a UltiMate 3000 RSLCnano system interfaced online to a Q Exactive HF Hybrid Quadrupole-Orbitrap MS instrument; the nano-LC system was equipped with an Acclaim PepMap 100 trapping column (75 µm × 2 cm) and 50-cm μPAC analytical column (all Thermo Fischer Scientific). Peptides were separated using a 75-min linear gradient (solvent A, 0.1% aqueous formic acid; solvent B, 0.1% formic acid in acetonitrile). Data were acquired in data-dependent acquisition mode using the Top20 method. The precursor *m*/*z* range was 350–1,600 Th, the resolution was 120,000 and 15,000 for precursor and fragments, respectively, and the dynamic exclusion time was set to 15 s. Acquired spectra were matched by Mascot software (version 2.2.04; Matrix Science) with a tolerance of 5 ppm and 0.025 Da for precursors and fragments, respectively, against *C*. *autoethanogenum* protein sequences in the National Center for Biotechnology Information (NCBI) database (August 2024, 23,655 entries); the results were evaluated by Scaffold software (version 5.3.3; Proteome Software) using 99% and 95% protein and peptide probability thresholds and a false discovery rate (Scaffold option) calculated at <1%. The raw MS data were deposited to the Edmond repository^56^.

### Estimation of electron content in the as-isolated *Ca*AFOR

The electron content of *Ca*AFOR was assessed by measuring the reduction of BV^2+^ during incubation with the as-isolated frozen *Ca*AFOR preparation. BV^2+^ was preferred to MV^2+^ because the higher redox potential of BV favors the electron transfer from the enzyme to the BV^2+^ molecule. The experiment was performed at 20 °C in an anaerobic chamber filled with an N_2_ (100%) atmosphere, in technical triplicate using a unique pool of enzyme with a protein concentration estimated at 7.93 g l^−1^ (119.54 µM). The enzyme was diluted with 25 mM Tris-HCl pH 7.6, 10% (v/v) glycerol and 2 mM DTT, supplemented or not with 25 mM BV^2+^, with a final *Ca*AFOR concentration of 102.46 µM and 3.57 mM BV^2+^. Absorbance spectra were monitored from 300 to 800 nm in anaerobic conditions in 384-well plates using a BMG Labtech FLUOstar Omega microplate reader. Spectra of buffer and buffer supplemented with BV^2+^ were also collected. By measuring the difference in absorbance at 600 nm between the AFOR supplemented or not with BV^2+^, it was estimated that 10.29 ± 3.35 µM electrons could be transferred from the *Ca*AFOR to BV^2+^ (using an experimentally determined molar extinction coefficient of 6,384.2 M^−1^ cm^−1^ for BV^+●^ for calculation).

### Sulfide quantification

The sulfide concentration in the dithionite solution was quantified by a protocol adapted from the diamine method^57^. Shortly, different volumes of a freshly prepared anoxic 5 mM sodium dithionite solution (in 50 mM Tris-HCl pH 8.0) were sampled and dissolved in aerobic 1 % (w/v) zinc acetate solution in glass tubes (final volume: 1 ml) to form zinc sulfide precipitates. Titration of sulfide was performed by the addition of 80 µl of the diamine reagent (4 g l^−1^ *N*,*N*-dimethyl-1,4-phenylenediamine sulfate and 6 g l^−1^ FeCl_2_∙H_2_O, dissolved in a cold 12 M HCl solution). The reaction was performed for 30 min in the dark before the spectrophotometric measurement of the reaction product at 670 nm. Sulfide concentration was determined by comparing it with the standard of an anoxic pure sulfide solution.

### Phylogeny analysis

The protein sequence of *Ca*AFOR was used as a query for BLAST^58^ by searching in the PDB and RefSeq database. The RefSeq database was selected to limit species redundancy and because it usually contains genomes of unquestionable quality. The searches in the RefSeq database were set up to extract 500 sequences. Sequences with more than 90% identity were removed using CD-HIT^59,60^, yielding 336 sequences. The sequences extracted from the PDB and from the work of Arndt et al.^12^ were added, leading to a total of 369 sequences used for the construction of the tree. The list of sequences used for the analysis is available from Zenodo^61^. The phylogenetic trees were constructed using the maximum-likelihood method and generated with the MEGA program^62^ using an alignment constructed with MUSCLE^63^. A total of 200 replicates were used to calculate each node score. The figure was constructed using iTOL^64^ and the taxonomy was performed using NCBI taxonomy^65^.

Supplementary Fig. 4a was constructed using the WebLogo 3 webserver^66^, from an alignment performed with Clustal Omega^67^ using the 336 sequences obtained by BLAST and CD-HIT.

Extended Data Fig. 8a was constructed using the genomic environment, putative operon and gene length predicted by the Operon Mapper server^68^ on the genome of *C*. *autoethanogenum*.

### Crystallization and structure determination

Crystallization was performed anaerobically by initial screening at 20 °C using the sitting-drop method on 96-Well MRC two-drop polystyrene crystallization plates (SWISSCI) in a Coy tent containing an N_2_/H_2_ (97:3) atmosphere. The reservoir chamber was filled with 90 μl of crystallization condition (SG1 crystallization screen from Molecular Dimensions) and the crystallization drop was formed by spotting 0.55 μl of purified protein with 0.55 μl of precipitant. The protein was crystallized at 8.6 mg ml^−1^ in storage buffer and the precipitant solution contained 200 mM MgCl_2_·6H_2_O, 100 mM Bis–Tris pH 6.5 and 25% (w/v) PEG3350. The crystals were soaked in the crystallization solution supplemented with 25% (v/v) glycerol for a few seconds before freezing in liquid nitrogen.

### Data collection and structural analysis

The diffraction experiments used for the deposited model were performed at 100 K on beamline PROXIMA-1 from SOLEIL. The initial determination of the structure was performed using diffraction data collected on beamline P11 from DESY. The data from SOLEIL were preferred for refinement on the basis of a lower pseudotranslational symmetry. Indeed, the analysis of the Patterson function by Xtriage revealed a notable off-origin peak that was 66.05% of the origin peak, indicating pseudotranslational symmetry. The diffraction data were deposited to Zenodo^69^. The data were processed and scaled with autoPROC^70^. The data presented anisotropy (along the following axes: *a* = 1.92 Å, *b* = 1.57 Å and *c* = 1.71 Å) and were further processed with STARANISO correction integrated with the autoPROC pipeline (http://staraniso.globalphasing.org/cgi-bin/staraniso.cgi; Global Phasing).

The structure was solved by processing a single-wavelength anomalous dispersion experiment at the W L3 edge (Supplementary Fig. 2 and Extended Data Table 1) using the CRANK-2 program. The model was manually built using Coot^71^ and refined with PHENIX (version 1.20.1-4487). The last refinement steps were performed by refining with a translation libration screw and models were validated by the MolProbity server^72^. The model was refined and deposited with hydrogens in the riding position. The structure was deposited to the PDB under accession code 9G7J. Data collection and refinement statistics for the deposited models are listed in Extended Data Table 1. All figures with structures were generated and rendered with PyMOL (version 2.2.0; Schrödinger).

The data presented in Fig. 1d were obtained from an analysis of the PDB. The shortest distance between the pterin and the closest [4Fe–4S] was measured and an average of the measures obtained in the asymmetric unit was calculated. This average was indicated in the figure (*Ca*AFOR) or used to calculate an average for the group of enzymes. The structures used were from the PDB under the following accession codes: (1) *Ca*AFOR (9G7J); (2) 1B25, 1AOR, 8C0Z, 6X1O, 6X6U and 4Z3W (aldehyde oxidases); (3) 5T5I, 2VPW, 1AA6, 7BKB, 7VW6, 7E5Z, 2E7Z, 5E7O, 4YDD, 2JIO, 2JIM, 3EGW, 3IR7, 1R27, 1Q16 and 3IR5 (bis-PGD enzymes) and (4) 5G5G, 5G5H, 5Y6Q, 1RM6, 4ZOH, 1DGJ, 1T3Q, 4C7Y, 1SIJ, 7OPN, 5EPG, 7DQX, 4UHW, 7PX0, 1N5W, 1ZXI and 8GY3 (xanthine oxidases).

The ferredoxin–AFOR complex structure prediction was performed by AlphaFold2 using the sequences of ferredoxin (WP_013236834) and AFOR (WP_013238665) from *C*. *autoethanogenum*. The five generated models were deposited to Zenodo^54^.

### Molecular docking

All molecular dockings were performed with the online Caver Web server (version 2.0)^73^ using the chain A of *Ca*AFOR as a template. The calculations were performed with all default parameters without molecular dynamics and by selecting an origin close to the oxo and hydroxo ligands. All ligands except waters (that is, including the oxo and hydroxo ligands) were considered for tunnel calculation and substrate diffusion. All dynamic simulation and binding energy profiles were deposited to Zenodo^74^.

### Native electrophoresis and size-exclusion chromatography

The high-resolution clear native PAGE (hrCN PAGE) protocol was adapted from Lemaire et al.^75^. Glycerol was added to the sample at a final amount of 20% (v/v). Ponceau S at a final concentration of 0.001% (w/v) served as a marker to follow the migration. The buffer composition for the electrophoresis cathode was 50 mM tricine, 15 mM Bis–Tris-HCl pH 7.0, 0.05 % (w/v) sodium deoxycholate, 2 mM DTT and 0.01 % (w/v) dodecyl maltoside, whereas the anode buffer contained 50 mM Bis–Tris-HCl pH 7.0 and 2 mM DTT. An 8–15% linear polyacrylamide gradient gel was used and electrophoresis was run under a N_2_/CO_2_ (90:10) atmosphere with a constant 40 mA of current (PowerPac basic power supply; Bio-Rad). After electrophoresis, protein bands were visualized with Ready Blue protein gel stain (Sigma-Aldrich). The native protein ladder used was NativeMark unstained protein standard (Thermo Fischer Scientific).

The determination of the oligomeric state by gel filtration was performed on Superdex 200 10/300 GL and Superose 6 Increase 10/300 GL (GE Healthcare) columns in 25 mM Tris-HCl pH 7.6, 10% (v/v) glycerol and 2 mM DTT at a flow rate of 0.4 ml min^−1^ in an anaerobic Coy tent containing an N_2_/H_2_ (97:3) atmosphere at 20 °C. A high molecular weight range gel filtration calibration kit (GE Healthcare) was used as the protein standard.

### Activity measurements

The enzyme preincubation for reactivation was performed in 50 mM Tris-HCl pH 8.0 at 20 °C in an anaerobic chamber filled with an N_2_ (100 %) atmosphere. The *Ca*AFOR final concentration in the preincubation mix ranged between 0.16 and 4.7 µM. The final concentration of ferredoxin used in the preincubation mix ranged from 0 to 250 µM. When indicated, 500 µM Ti(III) citrate, 500 µM ferricyanide, 500 µM dithionite and 500 µM sulfide (final concentration) in the preincubation mix were used.

The reactivation procedure showed some discrepancies in the obtained final activity of the enzyme (Extended Data Fig. 5c). This was indicated by performing different reactivation procedures on the same enzyme stock and measuring in triplicate the enzyme activity of each reactivated *Ca*AFOR batch. Therefore, to minimize this effect, the presented activities (displayed as the average and s.d.) were calculated from different measurements performed from distinct reactivation procedures prepared simultaneously. In other words, the replicates were measured using distinct reactivation tubes. This explains the relatively large s.d. in our results and allows us to incorporate the discrepancies of reactivation in the results. No standardization procedure was applied to these results. In the cases of Fig. 3c and Extended Data Fig. 5f, the measures obtained with different final ferredoxin concentrations were performed using the same replicates of reactivation, using different volumes of the reactivated enzyme in the assay. The only experiments performed with *Ca*AFOR that were not performed with a replicate of reactivation procedures are those displayed in Extended Data Fig. 5c (highlighting the reactivation discrepancies) and Supplementary Fig. 10, where triplicates were obtained from a single pool of reactivated enzyme.

Aldehyde:MV^2+^ oxidoreduction activity was assayed by measuring the reduction of MV^2+^ at 600 nm in 50 mM Tris-HCl pH 8.0 and 5 mM MV^2+^ at 37 °C in a BMG Labtech FLUOstar Omega microplate reader in an anaerobic chamber filled with an N_2_ (100%) atmosphere. When indicated, BV^2+^ was used instead of MV^2+^ at the same concentration. The final protein concentration ranged from 1.6 to 66 nM. An experimentally determined molar extinction coefficient of 17,213 M^−1^ cm^−1^ for MV^+●^ and 6,384.2 M^−1^ cm^−1^ for BV^+●^ was used for calculation. Freshly prepared solutions of aldehyde were used because of aldehyde instability.

As for other characterized aldehyde oxidases, BV^2+^ acts as a more suitable electron acceptor for aldehyde oxidation than MV^2+^ (Extended Data Fig. 5b). As the measured viologen-based activities were used to compare different conditions, the nature of the used artificial electron acceptor had no effect on the conclusions of this work, as the important stated activities were measured with the physiological electron shuttle ferredoxin.

All measurements were performed at least in triplicate, consisting of three different reaction mixtures using the same reactivated enzyme, and are presented in μmol of MV^2+^, BV^2+^ or ferredoxin reduced per min per mg of protein (as indicated). Alternatively, the assays are presented in NADH oxidized per min per mg of protein (as indicated). The data used for calculation were subtracted from the baseline measured without substrate addition. The kinetic parameters for formaldehyde could not be estimated as the necessary concentrations of the aldehyde triggered enzyme aggregation.

The effect of O_2_ on the preincubated *Ca*AFOR could not be assessed because of the complexity of the experimental setup (for example, cross-reaction with Ti(III) citrate, ferredoxin and sulfide).

The CO sensitivity was assayed using a Cary 60 ultraviolet–visible light spectrophotometer (Agilent Technologies) in a 600-µl quartz cuvette at 37 °C. The reactivation was initiated by acetaldehyde addition and CO was added after the initial measurement of the activity.

The activity to check whether *Ca*AFOR can perform acetate reduction were tested with viologens and ferredoxin reduced with Ti(III) citrate in similar conditions to those previously mentioned. However, no acetate-dependent oxidation of the viologen and ferredoxin could be detected.

Ferredoxin reduction by *Ca*AFOR and CODH/ACS was assayed in a quartz cuvette at 37 °C. The measurement was performed at 50 µM ferredoxin, monitoring the activity at 390 nm, considering a single electron transferred to ferredoxin and with an experimentally determined molar extinction coefficient of 11,095.4 M^−1^ cm^−1^ (obtained by subtraction of the absorbance of aerobically oxidized and dithionite-reduced ferredoxin).

Assays with gases (100% CO, 100% H_2_ or 10:90 CO/N_2_ mixture) were carried out in a 600-µl quartz cuvette containing a 400-µl reaction mixture. The gas (0.1 ml) was anaerobically injected with a syringe. The data used for calculation were subtracted from the baseline measured before gas addition.

Coupled enzymatic assays were measured in a quartz cuvette at 37 °C. Concentrations of *Ca*AFOR and CODH/ACS of 32 and 1,214 nM were used, respectively. A final concentration of ferredoxin ranging from 2 to 10 µM was used. The ethanol dehydrogenase from *S*. *cerevisiae* (15.6 U per ml final) was used to reduce acetaldehyde, with 0.2 mM NADH used as an electron donor. Oxidation of NADH was used to monitor activity, followed at 340 nm, calculated considering 1 mol of oxidized NADH per mol of reduced acetaldehyde and using an experimentally determined molar extinction coefficient of 5,297.9 M^−1^ cm^−1^ for calculation. The addition of 0.1 ml CO (100% or 10%) was used to initiate the reaction. The data used for calculation were subtracted from the baseline measured before CO addition. With the *Ca*CODH/ACS and ethanol dehydrogenase being in large excess, the measured NADH oxidation rate was considered equivalent to the acetate reduction by the *Ca*AFOR. Specific activity was calculated per mg of *Ca*AFOR and turnover was determined considering the *Ca*AFOR molecular weight.

### Reporting summary

Further information on research design is available in the Nature Portfolio Reporting Summary linked to this article.

## Online content

Any methods, additional references, Nature Portfolio reporting summaries, source data, extended data, supplementary information, acknowledgements, peer review information; details of author contributions and competing interests; and statements of data and code availability are available at 10.1038/s41589-025-02055-3.

## Supplementary information

Supplementary Information Supplementary Figs. 1–10, References and uncropped SDS–PAGE of Supplementary Fig. 1.

Reporting Summary

Supplementary Data 1 Source data files for the supplementary figures.

## Source data

Source Data Fig. 1 Datasets and calculations.

Source Data Fig. 3 Datasets and calculations.

Source Data Fig. 4 Datasets and calculations.

Source Data Extended Data Fig. 1 Datasets, calculations and gels.

Source Data Extended Data Fig. 4 Datasets and calculations.

Source Data Extended Data Fig. 5 Datasets and calculations.

Source Data Extended Data Fig. 6 Datasets and calculations.

Source Data Extended Data Fig. 7 Datasets and calculations.

## Figures and Tables

**Extended Data Table 1:**
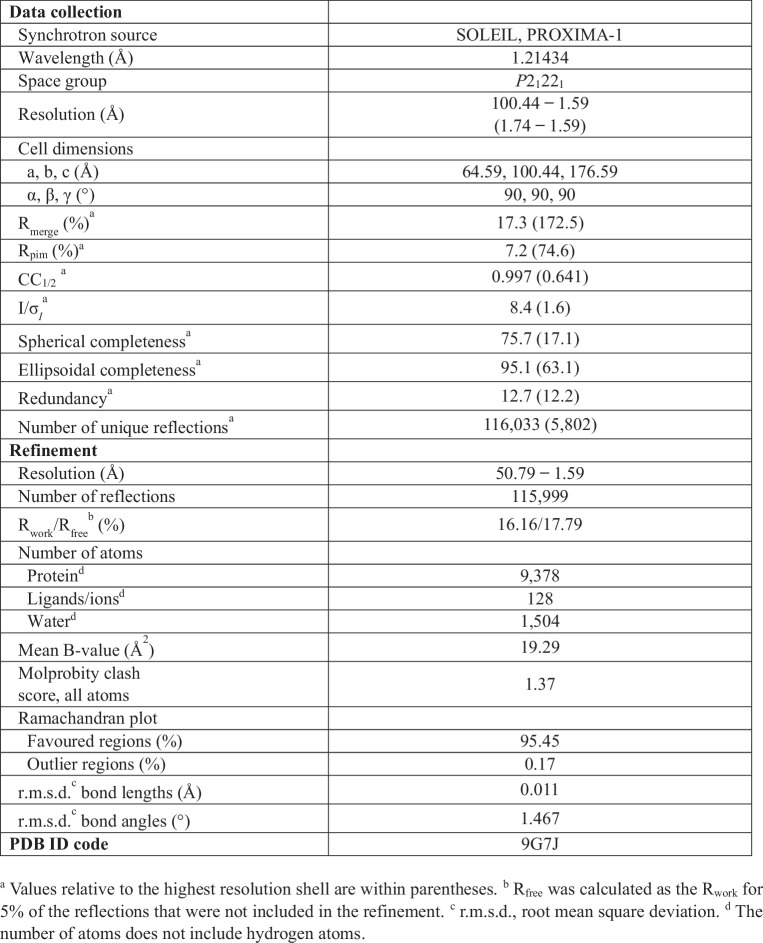
X-ray analysis statistics for the structure of *Ca*AFOR

## Data Availability

The *Ca*AFOR structure was validated and deposited to the PDB under accession number 9G7J. The raw X-ray diffraction data were deposited to Zenodo (10.5281/zenodo.16725608)^69^. The MS raw data generated in this study were deposited to the Edmond database (10.17617/3.AQASBK)^56^. The sequences used for phylogeny analysis and residue conservation study were deposited to Zenodo (10.5281/zenodo.15229728)^61^. The structural model of the complex of AFOR and ferredoxin from *C*. *autoethanogenum* was deposited to Zenodo (10.5281/zenodo.15083112)^54^. All dynamic simulation and binding energy profiles were deposited to Zenodo (10.5281/zenodo.15342502)^74^. All other data are available in the manuscript or the Supplementary Information. Source data are provided with this paper.
