# Supplementary material for: Carbon-monoxide-driven bioethanol production operates through a tungsten-dependent catalyst
Source: Nat Chem Biol. 2025 Oct 29;22(1):28–36. doi: 10.1038/s41589-025-02055-3 (PMC12727526; doi:10.1038/s41589-025-02055-3)

nature chemical biology

Supplementary information https://doi.org/10.1038/s41589-025-02055-3

Carbon-monoxide-driven bioethanol production operates through a tungsten-dependent catalyst

In the format provided by the authors and unedited

Supplementary Information

This file contains:

The Supplementary Data Fig. 1 to 10

The references present in the supplementary information

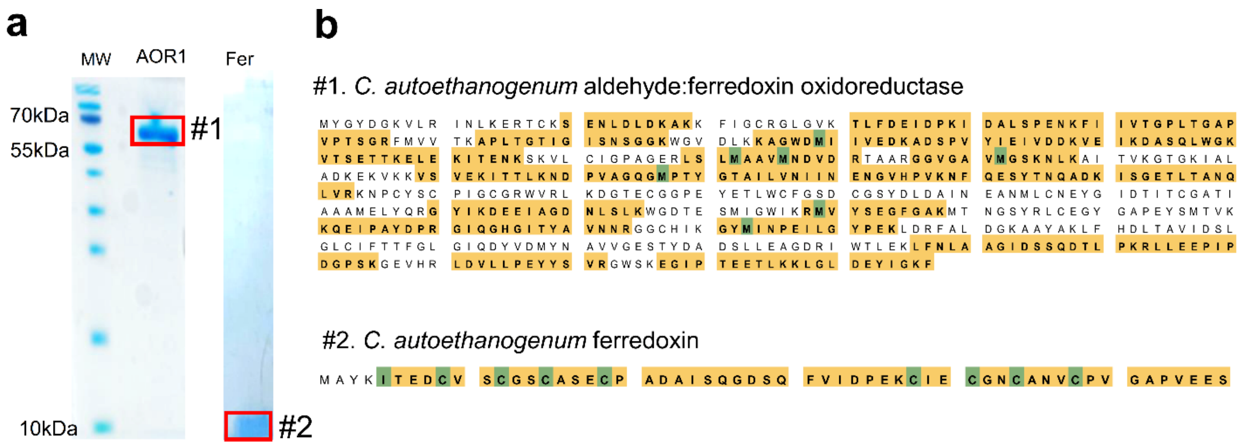

c

Sample	Protein name	Gene name	Acc. No.	Molecular weight	Mass Spectrometry Analysis matched		
					Number of unique peptides	Number of spectra	Sequence coverage
#1	Aldehyde:ferredoxin oxidoreductase*	*Aor1*	WP_013238665	66 kDa	37	225	55%
#2	Ferredoxin	*fer*	WP_013236834/ OVY50687.1	6 kDa	2	39	93%

Supplementary Data Fig. 1. Mass Spectrometric identification of AOR1 (*Ca*AFOR) and ferredoxin proteins purified from *C. autoethanogenum*. **a**, Coomassie-stained denaturing polyacrylamide gel electrophoresis (PAGE) of purified *Ca*AFOR and ferredoxin. Gel regions analysed by mass spectrometry are designated with red squares. Selected MS markers are shown on the left-hand side. **b**, Distribution of peptides detected by mass spectrometry for *Ca*AFOR and ferredoxin in samples #1 and #2. MS-matched peptides are highlighted in yellow, and amino acids detected carrying modifications (oxidation, carbamidomethylation, acetylation) are marked in green. **c**, MS-identification details for both proteins in the corresponding gel regions (a). * Only traces of the second AFOR isoform or *C. autoethanogenum* WP_013238675.1 (two peptides) were detected in the band #1.

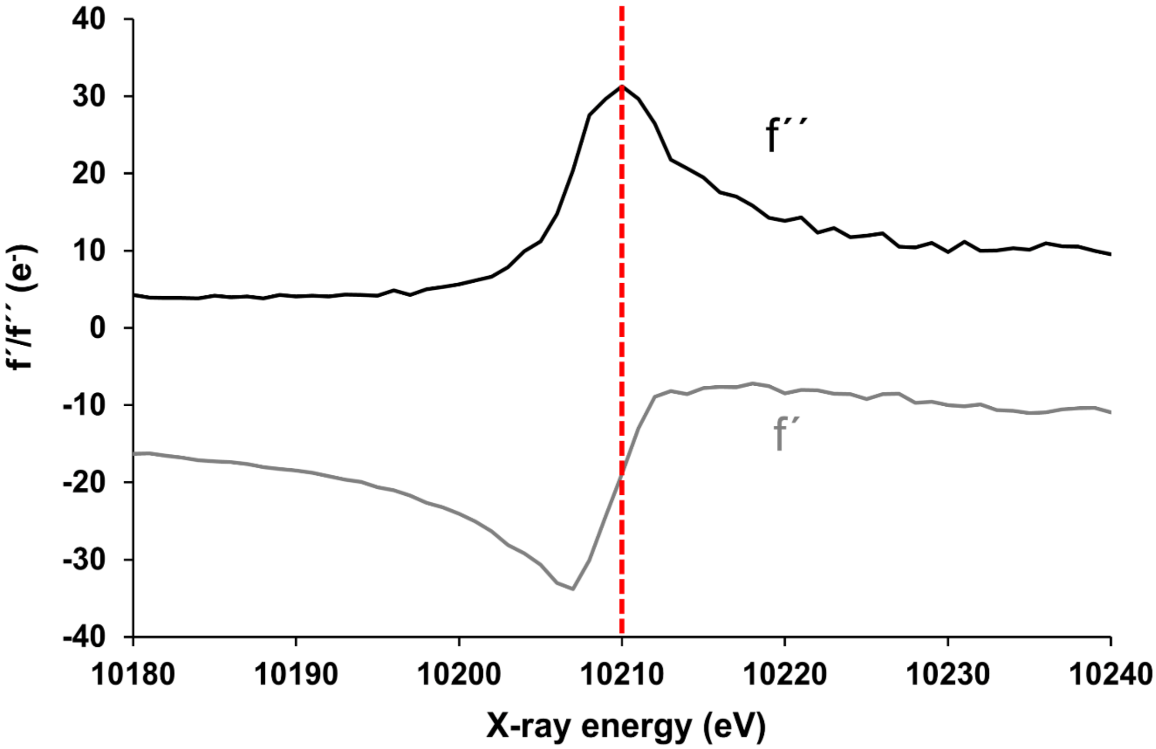

Supplementary Data Fig. 2. Fluorescence scan from a *Ca*AFOR protein crystal. The fluorescence counts are given as a function of energy (eV). The dashed red line indicates the energy used for data collection (10,210 eV).

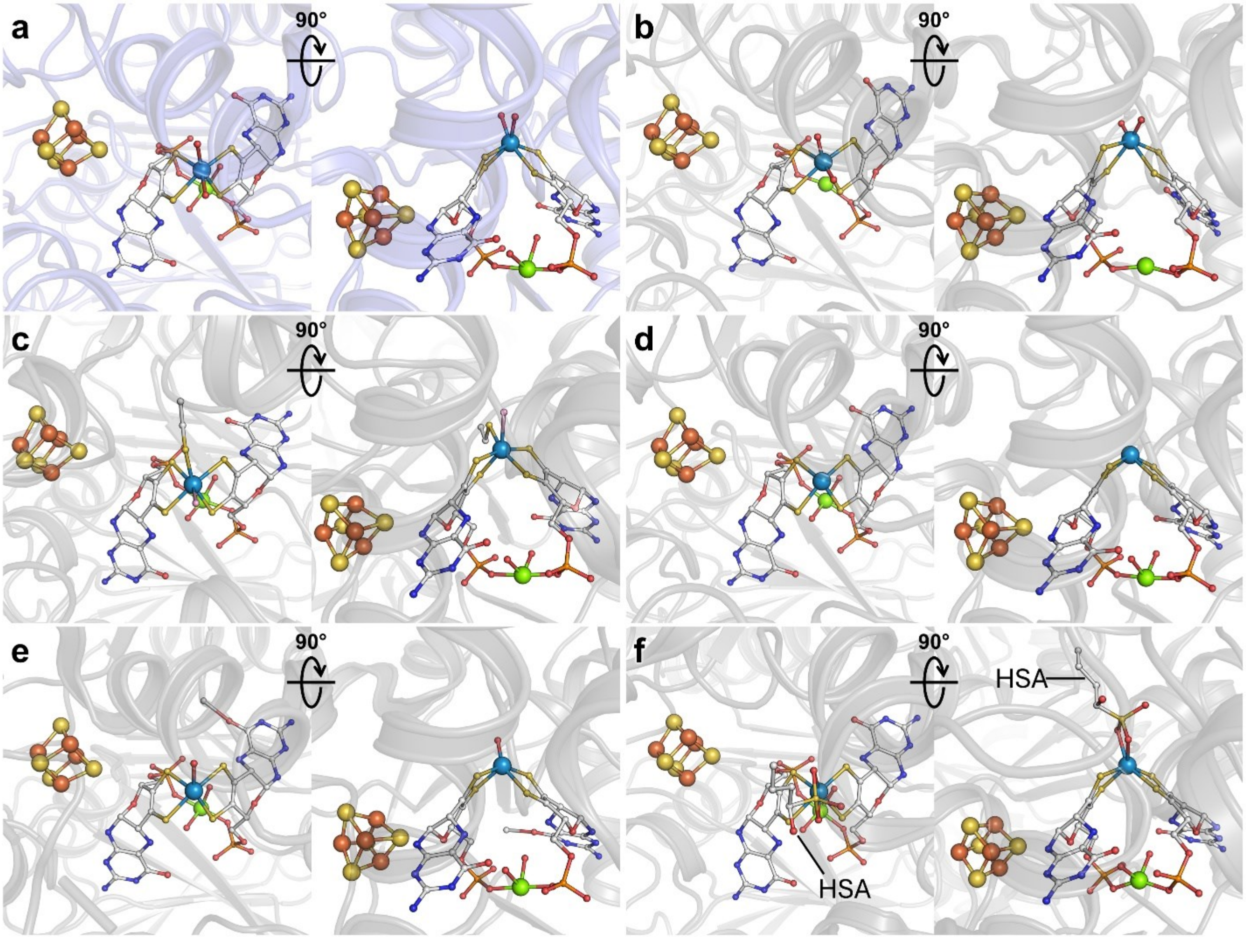

Supplementary Data Fig. 3. Cofactor organisation in *Ca*AFOR structure and homologs. The structure of *Ca*AFOR (**a**, PDB 9G7J), *Aa*AOX (**b**, PDB 8C0Z), *Gm*BamB (**c**, PDB 4Z3W), *Pf*AFOR (**d**, PDB 1AOR), *Pf*FFOR (**e**, PDB 1B25), and *Pf*ASOR (**f**, PDB 6X1O) are shown in two different orientations. Protein chains are shown as cartoons (coloured blue for *Ca*AFOR, grey for the others). Cofactors, substrates, and the residues part of the W coordination are shown as balls and sticks, with carbon, oxygen, nitrogen, sulfur, phosphorus, magnesium, iron, and tungsten are coloured white, red, blue, light yellow, light orange, green, orange and grey blue, respectively. HSA stands for (1R)-1-hydroxybutane-1-sulfonic acid, which is found in the *Pf*ASOR structure.

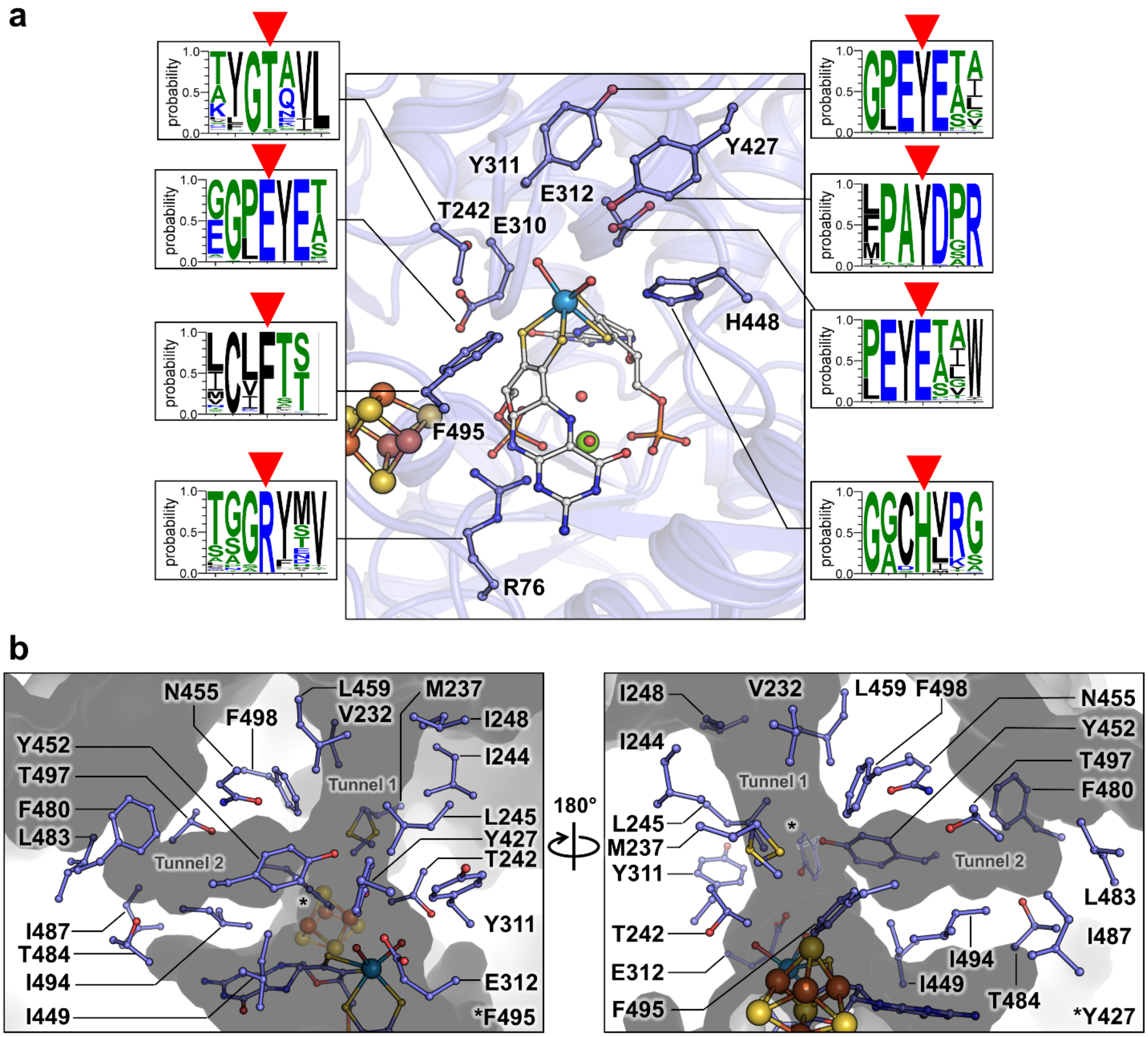

Supplementary Data Fig. 4. Residue conservation in the vicinity of the catalytic tungsten atom. a, The structure of *Ca*AFOR is shown as a cartoon with cofactors and neighbouring residues shown as balls and sticks. Schematic representation of residue conservation (constructed with WebLogo 3[1]) using the 336 sequences extracted from the RefSeq database (Extended Data Fig. 2) is shown in the side panels (therefore excluding the sequences extracted from the PDB and from the work from Arndt et al. 2019 as described in the Materials and Methods). A red arrow indicates the residue of interest in each panel. **b**, Access to the active site. Two different orientations showing the two tunnels connecting the active sites are shown. The protein is shown as a grey surface. The residues coordinating the tunnels leading to the active sites are shown as balls and sticks. **a** and **b**, Oxygen, nitrogen, sulfur, phosphorus, magnesium, iron, and tungsten are coloured red, blue, light yellow, light orange, green, orange and grey blue, respectively.

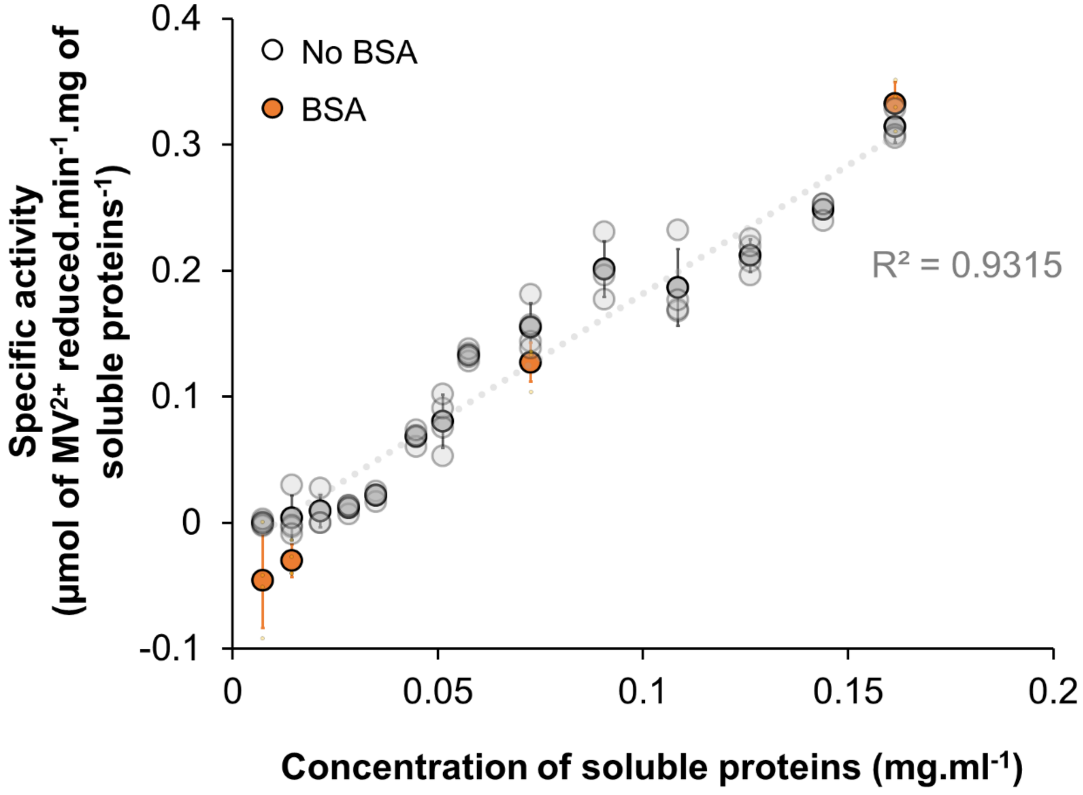

Supplementary Data Fig. 5. AFOR specific activity in soluble extracts depending on the extract concentration. The activity was measured in the presence (orange circles) or the absence (white circles) of bovine serum albumin (BSA). Averages and standard deviations are shown, with individual data shown as transparent grey dots. The measurements were done in replicates (n=3 or 4), from independent treatments of different dilutions of the same enzyme pool. MV^{2+} is used as an electron acceptor.

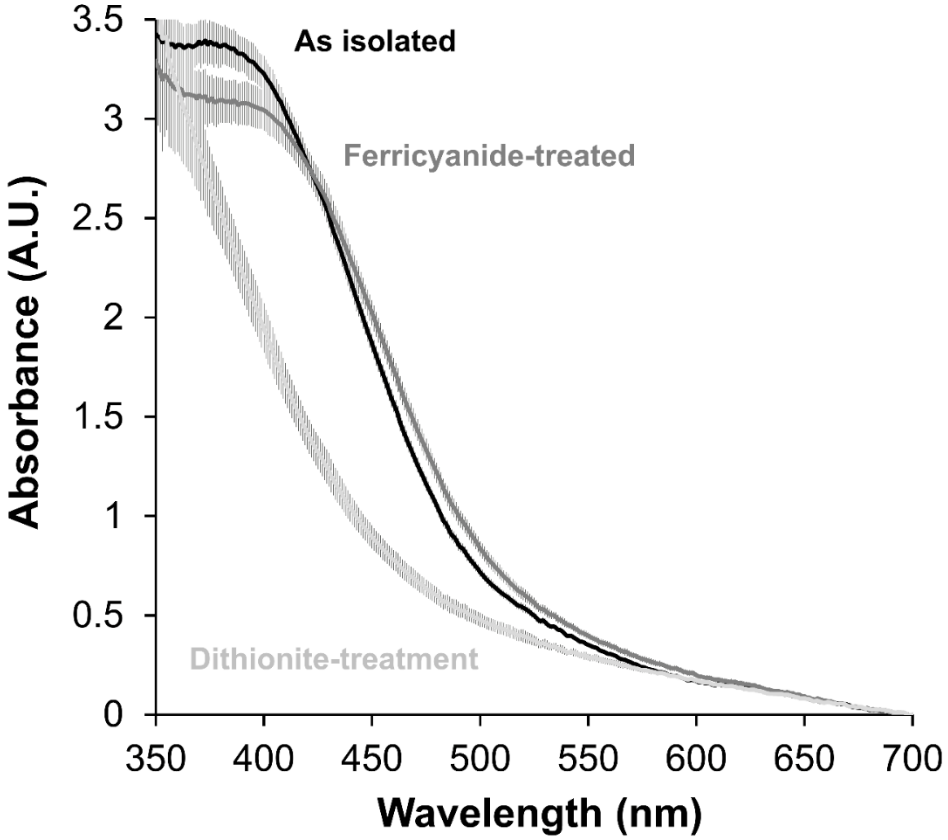

Supplementary Data Fig. 6. Redox state of the isolated ferredoxin. The absorbance spectrum of the ferredoxin as isolated or treated with an oxidising (ferricyanide) or reducing (dithionite) agent. Average and standard deviation of three distinct spectra are displayed. The curves were normalized by subtractions of the absorbance values by the absorbance at 700 nm for each curve.

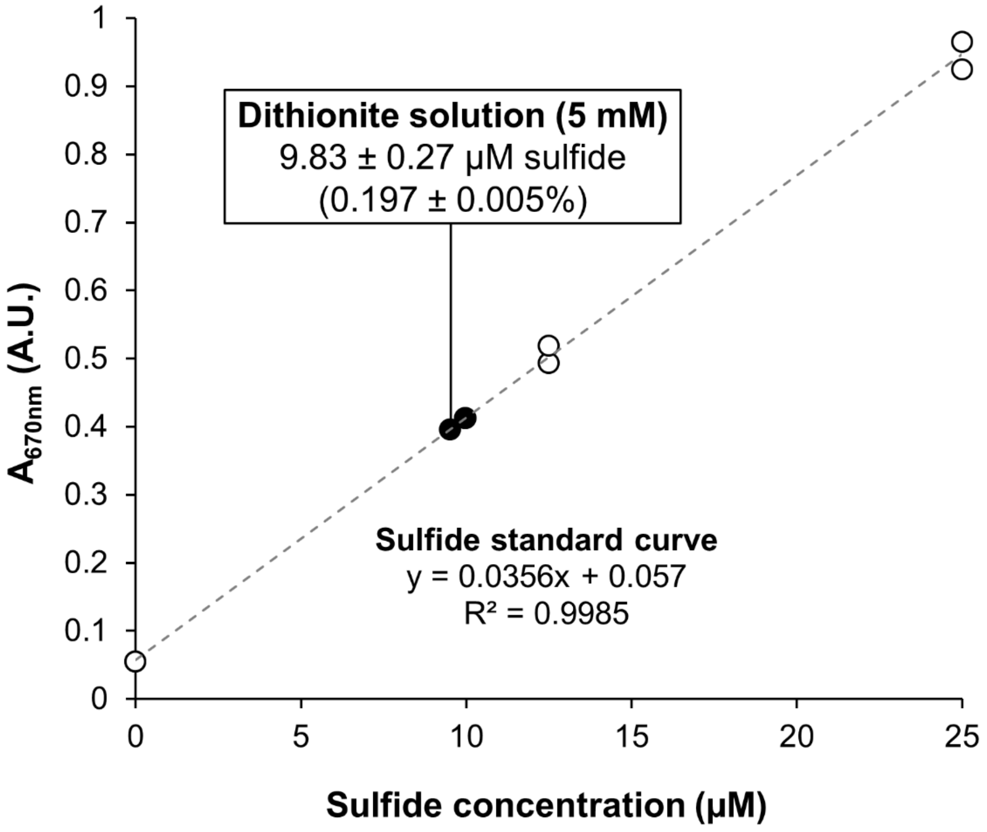

Supplementary Data Fig. 7. Sulfide quantification in the dithionite solution used in the assay (Extended Data Fig. 5a). Absorbance at 670 nm is plotted. Measures of the sulfide standards and dithionite samples (n=3) are shown as white and black circles, respectively.

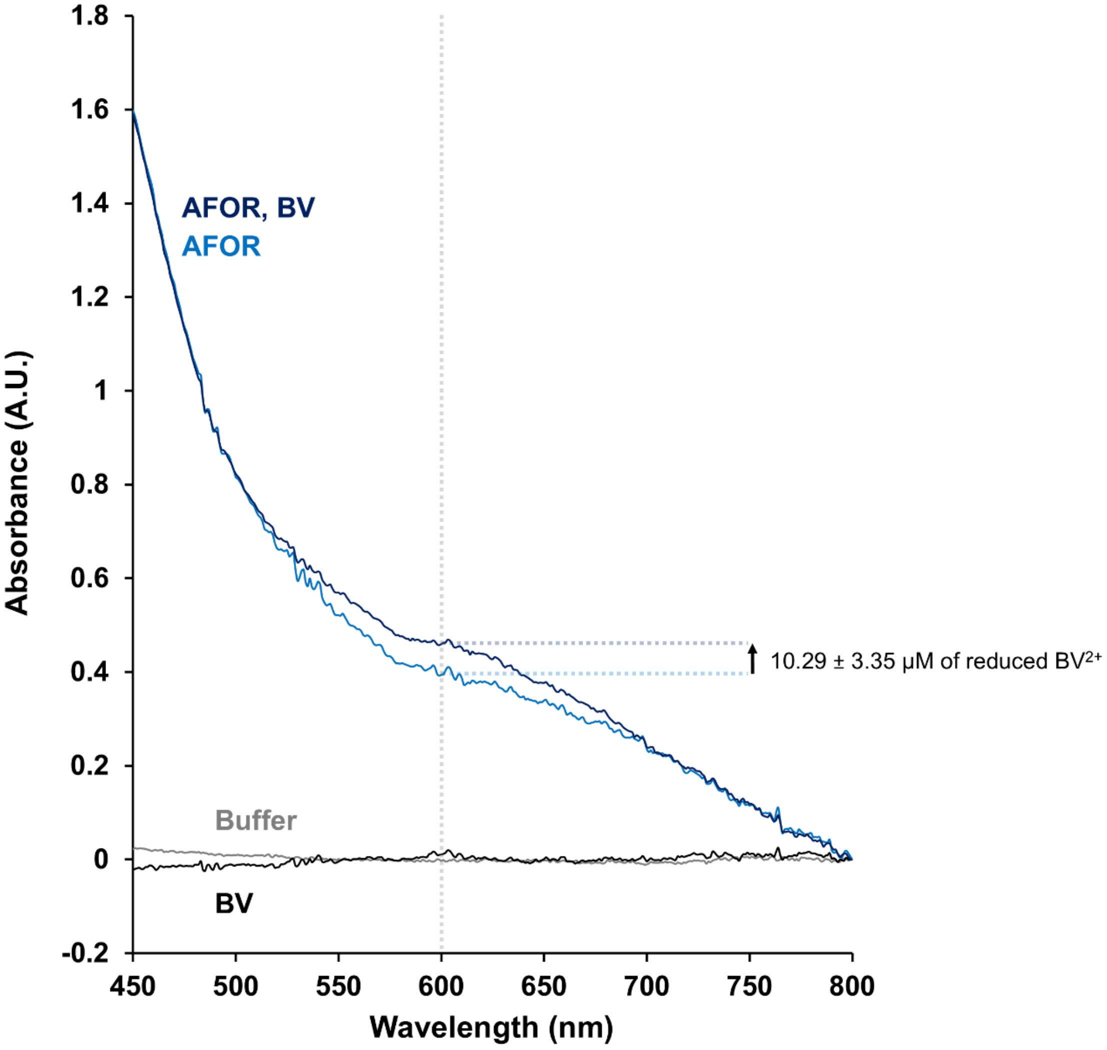

Supplementary Data Fig. 8. Estimation of the electron content of the as-isolated *Ca*AFOR. The absorbance spectra of buffer (grey), buffer supplemented with BV^{2+} (black), *Ca*AFOR (blue), and *Ca*AFOR supplemented with BV^{2+} (dark blue). The presented curves are averages of technical triplicates. No baseline correction was applied, but instead the curves were normalized by subtracting each value of a curve by the absorbance value at 800 nm. The number of transferred electrons was estimated by calculating the difference in absorbance at 600 nm in each replicate and determining the corresponding amount of reduced BV^{2+}, assuming a single electron per BV^{2+} reduced and a molar extinction coefficient of 6,384.2 $M^{-1}.cm^{-1}$.

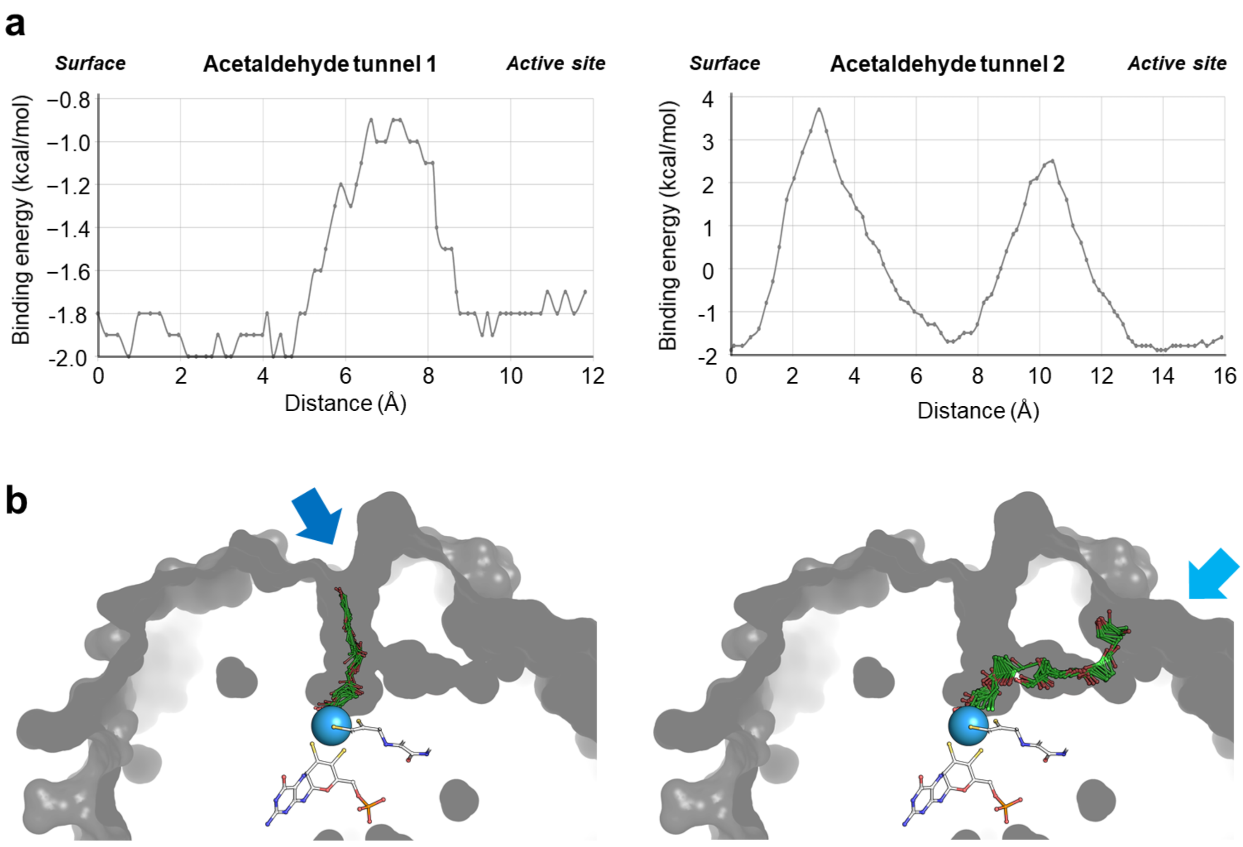

c

	Tunnel 1		Tunnel 2	
Docked molecules	Maximum binding energy (kcal/mol)	Minimum binding energy (kcal/mol)	Maximum binding energy (kcal/mol)	Minimum binding energy (kcal/mol)
**Acetaldehyde**	**−0.8**	**−2.0**	**3.8**	**−1.9**
Acetate	−0.5	−2.3	4.7	−2.3
Acetic acid	−0.8	−2.6	4.2	−2.6
Formaldehyde	−0.6	−1.2	2.4	−1.2
Proprionaldehyde	−1.5	−2.6	3.6	−2.5
Butyraldehyde	−1.9	−3.1	3.0	−2.8
(S)-acetoin	−0.9	−3.3	6.1	−3.2
(R)-acetoin	−0.9	−3.3	6.4	−3.3

Supplementary Data Fig. 9. Substrate docking based on CAVER Web analyses[2]. a, Comparison of the binding energy profiles of acetaldehyde between tunnel 1 and 2. The direction of acetaldehyde is from the surface to the active site. **b**, Illustration of the acetaldehyde path in *Ca*AFOR from the surface to the active site. The diffusion is compared between tunnel 1 (left, with the entrance highlighted by a blue arrow) and tunnel 2 (right, with the entrance highlighted by a cyan arrow). *Ca*AFOR surface and cavities are displayed by a grey surface with the tungstopterin and acetaldehyde shown as sticks. Tungsten is shown as a ball. Oxygen, nitrogen, sulfur, phosphorus, magnesium, iron, and tungsten are coloured red, blue, light yellow, light orange, green, orange and grey blue, respectively. **c**, Table indicating the different binding energy maxima and minima determined for the different substrates.

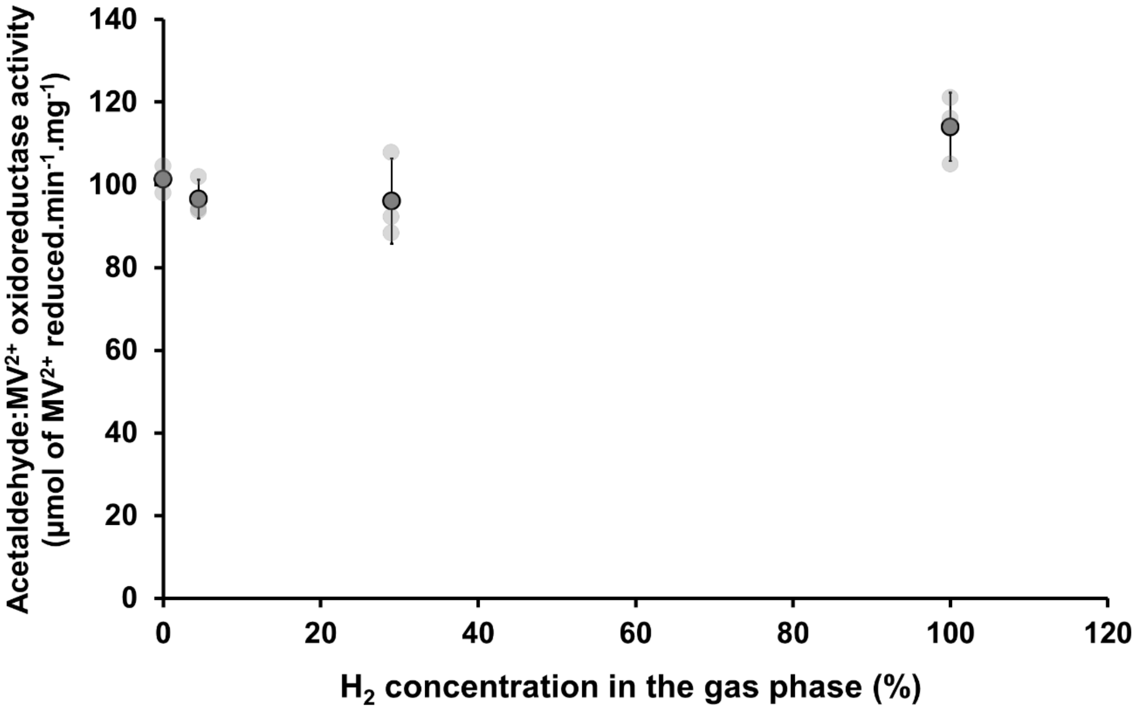

Supplementary Data Fig. 10. H_2 effect on the activity of *Ca*AFOR. Aldehyde: MV^{2+} oxidoreductase activity of *Ca*AFOR in the presence of H_2. The reactivated enzyme was first incubated in the presence of H_2, and the reactivation was initiated by the addition of acetaldehyde. Average and standard deviation are shown, with individual data shown as transparent grey dots. The measurements were done in replicates (n=3), from independent treatments of the same enzyme pool.

References.

1 Crooks, G. E., Hon, G., Chandonia, J. M. & Brenner, S. E. WebLogo: a sequence logo generator. *Genome Research* **14**, 1188-1190, doi:10.1101/gr.849004 (2004).

2 Stourac, J. *et al.* Caver Web 1.0: identification of tunnels and channels in proteins and analysis of ligand transport. *Nucleic Acids Research* **47**, W414-W422, doi:10.1093/nar/gkz378 (2019).

Uncropped gels used for Supplementary Data Figure 1

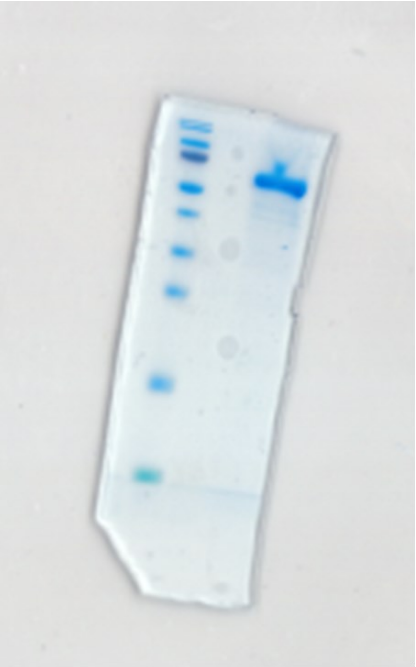

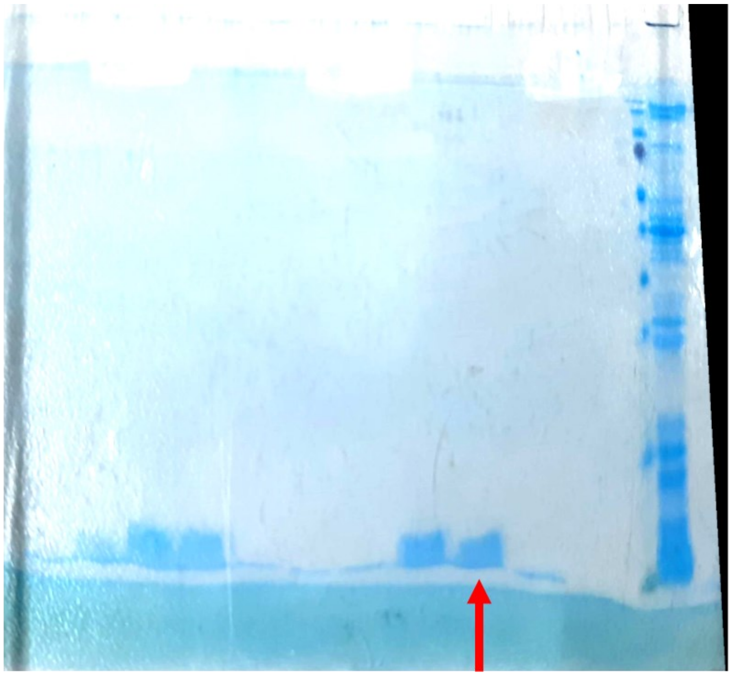

Supplement: Supplementary file 1 — Supplementary Figs. 1–10, References and uncropped SDS–PAGE of Supplementary Fig. 1. [file 41589_2025_2055_MOESM1_ESM.pdf]
