# Supplementary material for: Carbon-monoxide-driven bioethanol production operates through a tungsten-dependent catalyst
Source: Nat Chem Biol. 2025 Oct 29;22(1):28–36. doi: 10.1038/s41589-025-02055-3 (PMC12727526; doi:10.1038/s41589-025-02055-3)
Supplement: Supplementary file 2 — Reporting Summary [file 41589_2025_2055_MOESM2_ESM.pdf]

nature portfolio

Corresponding author(s): Olivier N Lemaire
Tristan Wagner

Last updated by author(s): Aug 7, 2025

Reporting Summary

Nature Portfolio wishes to improve the reproducibility of the work that we publish. This form provides structure for consistency and transparency in reporting. For further information on Nature Portfolio policies, see our Editorial Policies and the Editorial Policy Checklist.

Statistics

For all statistical analyses, confirm that the following items are present in the figure legend, table legend, main text, or Methods section.

n/a	Confirmed	
☐	☒	The exact sample size (*n*) for each experimental group/condition, given as a discrete number and unit of measurement
☐	☒	A statement on whether measurements were taken from distinct samples or whether the same sample was measured repeatedly
☒	☐	The statistical test(s) used AND whether they are one- or two-sided<br>*Only common tests should be described solely by name; describe more complex techniques in the Methods section.*
☐	☒	A description of all covariates tested
☒	☐	A description of any assumptions or corrections, such as tests of normality and adjustment for multiple comparisons
☐	☒	A full description of the statistical parameters including central tendency (e.g. means) or other basic estimates (e.g. regression coefficient) AND variation (e.g. standard deviation) or associated estimates of uncertainty (e.g. confidence intervals)
☒	☐	For null hypothesis testing, the test statistic (e.g. *F*, *t*, *r*) with confidence intervals, effect sizes, degrees of freedom and *P* value noted<br>*Give P values as exact values whenever suitable.*
☒	☐	For Bayesian analysis, information on the choice of priors and Markov chain Monte Carlo settings
☒	☐	For hierarchical and complex designs, identification of the appropriate level for tests and full reporting of outcomes
☒	☐	Estimates of effect sizes (e.g. Cohen's *d*, Pearson's *r*), indicating how they were calculated

Our web collection on statistics for biologists contains articles on many of the points above.

Software and code

Policy information about availability of computer code

Data collection	Spectrophotometer for activity measurement: Cary 60 UV-Vis Spectrophotometer Agilent technologies.<br>Microplate reader: BMG Labtech FLUOstar Omega Microplate reader.<br>Synchrotron data collection are all stated in the Extended Data Table 1.<br>Mass spectrometry: Orbitrap HF hybrid mass spectrometer (Thermo Fisher)
Data analysis	Microsoft Office Excel Professional Plus 2016 (16.0.5448.1000)<br>Microsoft Office Powerpoint Professional Plus 2016 (16.0.4266.1001)<br>MEGA X version 11.0<br>autoPROC 1.0.5<br>CCP4 package 8.0.004<br>PHENIX 1.20.1_4487<br>COOT version 0.9.8.3 EL<br>Open-source Pymol Version 2.2.0<br>Mascot software (v.2.2.04, Matrix Science, UK)

For manuscripts utilizing custom algorithms or software that are central to the research but not yet described in published literature, software must be made available to editors and reviewers. We strongly encourage code deposition in a community repository (e.g. GitHub). See the Nature Portfolio guidelines for submitting code & software for further information.

Data

Policy information about availability of data

All manuscripts must include a data availability statement. This statement should provide the following information, where applicable:

- Accession codes, unique identifiers, or web links for publicly available datasets
- A description of any restrictions on data availability
- For clinical datasets or third party data, please ensure that the statement adheres to our policy

The CaAFOR structure was validated and deposited in the Protein Data Bank (PDB) under the following accession number: 9G7J 77. The diffraction data were deposited on the Zenodo public repository 69. All other data were available in the manuscript or the supplementary materials. Source data files are provided in this work. The mass spectrometry raw data generated in this study have been deposited on the Edmund database 56. The sequences used for phylogeny analysis and residue conservation study have been deposited in the Zenodo 61. The structural model of the complex of the AFOR and ferredoxin from C. autoethanogenum has been deposited on Zenodo 54. All dynamic simulation and binding energy profiles are deposited on Zenodo 75.

Human research participants

Policy information about studies involving human research participants and Sex and Gender in Research.

Reporting on sex and gender	n.a.
Population characteristics	n.a.
Recruitment	n.a.
Ethics oversight	n.a.

Note that full information on the approval of the study protocol must also be provided in the manuscript.

Field-specific reporting

Please select the one below that is the best fit for your research. If you are not sure, read the appropriate sections before making your selection.

☒ Life sciences ☐ Behavioural & social sciences ☐ Ecological, evolutionary & environmental sciences

For a reference copy of the document with all sections, see nature.com/documents/nr-reporting-summary-flat.pdf

Life sciences study design

All studies must disclose on these points even when the disclosure is negative.

Sample size	The sample size for measurements has been stated in the material and methods section. Protein purification was performed multiple times (more than 5) with a similar, reproducible protocol. Experiments were performed with different samples of purified enzyme; yielding similar results. The exact number of replicates for each given value can be found in the Source Data file.
Data exclusions	Data were excluded only when there were evident experimental problems (e.g. oxygen contamination) or where controls indicated experimental flaws.
Replication	The activity measurements were performed at least in triplicates from enzymes purified from a single purification process. The exact number of replicates for each given value can be found in the Source Data file. The presented data are representative of several distinct purification processes.
Randomization	Randomization were applied to generate the Rfree set for the protein refinement. Except for structural analyses, randomization was not applied.
Blinding	Not relevant to the study.

Reporting for specific materials, systems and methods

We require information from authors about some types of materials, experimental systems and methods used in many studies. Here, indicate whether each material, system or method listed is relevant to your study. If you are not sure if a list item applies to your research, read the appropriate section before selecting a response.

Materials & experimental systems

n/a	Involved in the study
☒	☐ Antibodies
☒	☐ Eukaryotic cell lines
☒	☐ Palaeontology and archaeology
☒	☐ Animals and other organisms
☒	☐ Clinical data
☒	☐ Dual use research of concern

Methods

n/a	Involved in the study
☒	☐ ChIP-seq
☒	☐ Flow cytometry
☒	☐ MRI-based neuroimaging
